# USP21-EGFR-Lyn axis drives NSCLC progression and therapeutic potential of USP21 inhibition

**DOI:** 10.1186/s40364-025-00806-x

**Published:** 2025-07-09

**Authors:** Ji Hye Shin, Ji Young Kim, Mi-Jeong Kim, Yeeun Kang, Bongkum Choi, Dohee Kwon, Yoolim Sung, Seo Hyun Kim, Ha-Jeong Lee, Chaeeun Lee, Kyeong Kyu Kim, Jae-Hyuck Shim, Duk-Hwan Kim, Eunyoung Chun, Ki-Young Lee

**Affiliations:** 1 Department of Immunology, Samsung Biomedical Research Institute, Sungkyunkwan University School of Medicine, Suwon, Republic of Korea; 2 Division of Rheumatology, Department of Medicine, University of Massachusetts Chan Medical School, Worcester, MA USA; 3 Horae Gene Therapy Center, University of Massachusetts Chan Medical School, Worcester, MA USA; 4 Department of Medicine, Sungkyunkwan University School of Medicine, Suwon, Republic of Korea; 5 Bioanalysis Center, GenNBio Inc, Seongnam, Republic of Korea; 6 Department of Metabiohealth, Sungkyun Convergence Institute, Sungkyunkwan University, Suwon, Republic of Korea; 7 Department of Health Science and Technology, Samsung Medical Center, Samsung Advanced Institute for Health Science and Technology, Sungkyunkwan University School of Medicine, Seoul, Republic of Korea; 8 Department of Precision Medicine, Sungkyunkwan University School of Medicine, Suwon, Republic of Korea; 9 Research and Development Center, CHA Vaccine Institute, Seongnam, Republic of Korea

**Keywords:** Non-small cell lung cancer, Epidermal growth factor receptor, Ubiquitin specific peptidase 21, Lck/Yes novel tyrosine kinase, BAY-805

## Abstract

Non-small cell lung cancer (NSCLC) is a highly aggressive malignancy frequently driven by oncogenic mutations in the epidermal growth factor receptor (EGFR). Although EGFR-tyrosine kinase inhibitors (EGFR-TKIs) have shown clinical efficacy, challenges such as limited response duration and intrinsic mechanisms—such as EGFR amplification—can affect therapeutic outcomes. This study investigates the role of the USP21–EGFR–Lyn axis in NSCLC progression, identifying USP21 as a key regulator of EGFR and Lyn stability. Gene Set Enrichment Analysis (GSEA) of NSCLC patient datasets revealed a strong correlation between USP21 overexpression and poor prognosis. Functional studies using *USP21*-knockout (*USP21*-KO) lung cancer cell lines demonstrated reduced proliferation, migration, colony formation, and tumor spheroid growth. Mechanistically, USP21 interacts with EGFR and Lyn, preventing their ubiquitination and degradation, thereby sustaining oncogenic signaling. In vivo, USP21 depletion significantly suppressed tumor growth in xenograft models. Additionally, pharmacological inhibition of USP21 with BAY-805 effectively reduced EGF-induced tumor spheroid formation, highlighting its therapeutic potential. Collectively, these findings position USP21 as a promising target for NSCLC treatment and offer a potential approach to complement existing EGFR-targeted therapies.

**Supplementary Information:**

The online version contains supplementary material available at 10.1186/s40364-025-00806-x.

To the Editor,

Non-small cell lung cancer (NSCLC) is a highly aggressive malignancy frequently driven by oncogenic mutations in the epidermal growth factor receptor (EGFR) [1, 2]. Resistance to EGFR-tyrosine kinase inhibitors (EGFR-TKIs) remains a major challenge in clinical treatment [3, 4], with amplification of EGFR wild-type alleles identified as a contributing factor [5]. This study investigates the role of the USP21-EGFR-Lyn axis in NSCLC progression, highlighting USP21 as a key regulator of EGFR and Lyn stability and a potential biomarker for patient stratification.

To evaluate the clinical significance of USP21, we analyzed a microarray dataset from 42 NSCLC patients, which included lung tumor tissues (LTTs, *n* = 42) and matched lung normal tissues (mLNTs, *n* = 42) (Table S1, Fig. 1A). Based on differential expression magnitude (△Mag) of genes between LTTs and mLNTs, patients were stratified, and association and functional studies were conducted, as illustrated in Fig. 1A. Patients were categorized into two groups: USP21-upregulated (USP21^up^, *n* = 32) and USP21-downregulated (USP21^down^, *n* = 10) (Fig. 1B-C, Table S1). USP21^up^ patients showed enrichment in nine cancer-related gene sets (Fig. S1A-I). To explore the functional role of USP21, we generated three *USP21*-knockout (*USP21*-KO) lung cancer cell lines using CRISPR-Cas9 (Fig. 1D, Fig. S2A-C) [6]. Compared to control (Ctrl) cells, *USP21*-KO cells demonstrated significantly reduced proliferation, migration, colony formation, and 3D spheroid formation (Fig. 1E-G, Fig. S3A-M, Fig. S4A-B) [6–9]. In an in vivo model, tumor growth was markedly suppressed in NSG mice xenografted with *USP21*-KO H1299 cells compared to Ctrl H1299 xenografts (Fig. 1H-I).

**Fig. 1:**
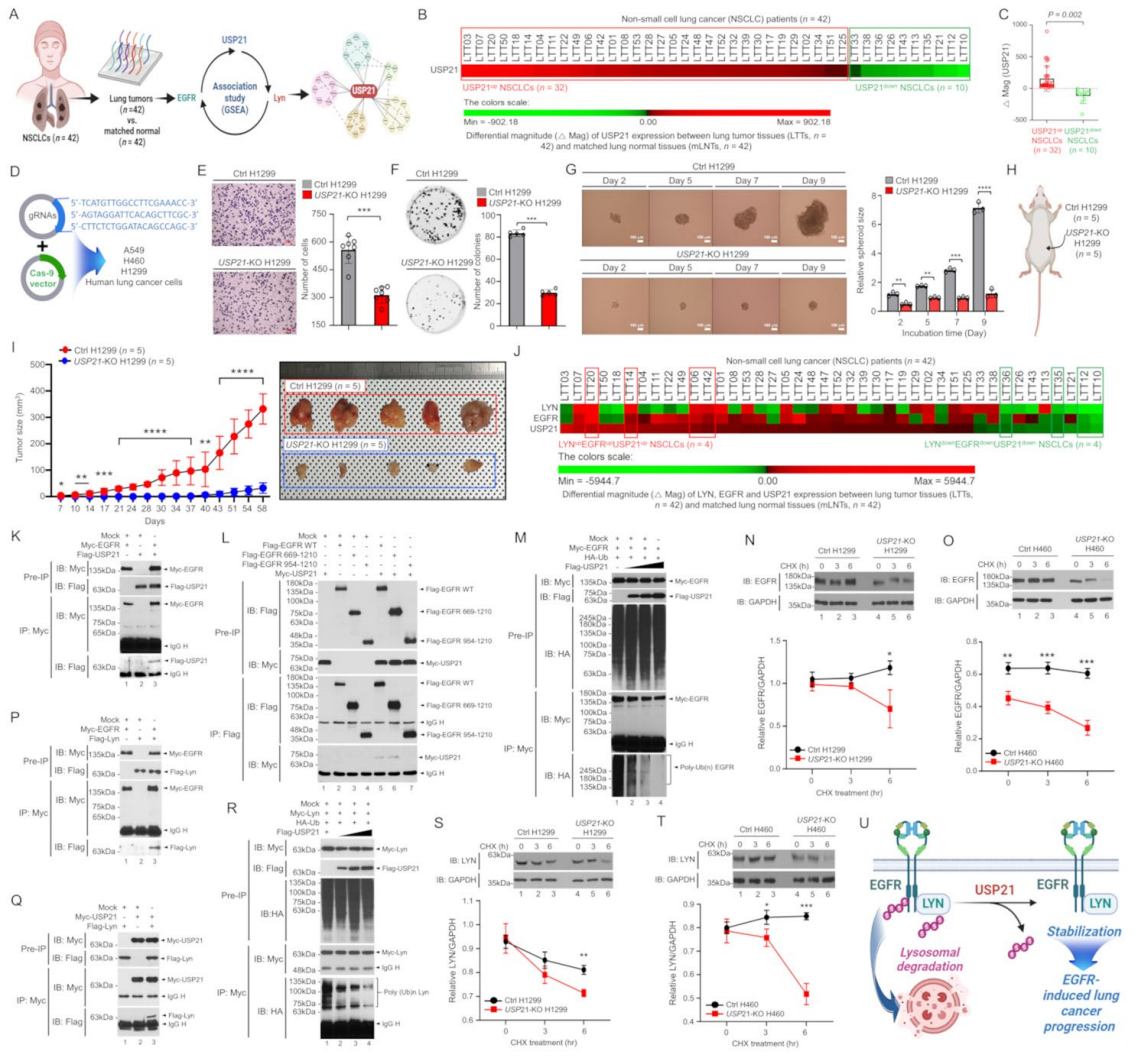
The USP21-EGFR-Lyn axis regulates lung cancer progression. **A** Schematic representation of the experimental workflow used to analyze associations and functional roles of USP21 in non-small cell lung cancer (NSCLC) patients (*n* = 42). **B** Differential expression magnitude (△Mag) of USP21 between lung tumor tissues (LTTs, *n* = 42) and matched lung normal tissues (mLNTs, *n* = 42). **C** △Mag of USP21 between USP21^up^ NSCLCs (*n* = 32) and USP21^down^ NSCLCs (*n* = 10). **D** CRISPR-Cas9 gene editing strategy for generating *USP21*-knockout (*USP21*-KO) lung cancer cells using guide RNAs targeting USP21. **E** Transwell migration assay was performed on control (Ctrl) H1299 cells and *USP21*-KO H1299 cells. Results are presented as mean ± SD (*n* = 7). Statistical significance (Student’s t-test); ***, *P* < 0.001. **F** Anchorage-dependent colony formation assay conducted with Ctrl H1299 and *USP21*-KO H1299 cells. Results are presented as mean ± SD (*n* = 5). Statistical significance (Student’s t-test); ***, *P* < 0.001. **G** 3D tumor spheroid formation assay was performed with Ctrl H1299 and *USP21*-KO H1299 cells. Results are presented as mean ± SD (*n* = 3). Statistical significance (Student’s t-test); **, *P* < 0.01; ***, *P* < 0.001; ****, *P* < 0.0001. **H** and **I** Xenograft mouse model: NSG mice were subcutaneously injected with either Ctrl H1299 (5 × 10⁶ cells per mouse, *n* = 5) or *USP21*-KO H1299 cells (5 × 10⁶ cells per mouse, *n* = 5) (**H**). Tumor volume was measured using calipers for up to 58 days post-injection. Tumor volumes (mm³) were calculated as (length × width²) × 0.5. Tumor growth curves are shown as mean ± SEM (**I**, left). Representative tumor-bearing NSG mice are shown at day 58 post-injection (**I**, right). Statistical significance: *, *P* < 0.05; **, *P* < 0.01; ***, *P* < 0.001; ****, *P* < 0.0001. **J** Differential expression magnitude (△Mag) of Lyn, EGFR, and USP21 between LTTs (*n* = 42) and matched LNTs (*n* = 42). **K-M** Co-immunoprecipitation (IP) and immunoblotting (IB) analyses in HEK-293T cells transfected with the indicated constructs: (**K**) IP using an anti-Myc antibody, followed by IB with anti-Flag or anti-Myc antibodies. (**L**) IP using an anti-Flag antibody, followed by IB with anti-Flag or anti-Myc antibodies. (**M**) IP using an anti-Myc antibody, followed by IB with anti-Flag, anti-Myc, or anti-HA antibodies. **N** and **O** Western blot analysis of EGFR degradation in Ctrl and *USP21*-KO lung cancer cells (H1299 in **N**, H460 in **O**) treated with cycloheximide at the indicated time points. EGFR degradation was quantified relative to GAPDH using ImageJ. Data are presented as mean ± SD from three independent experiments. Statistical significance (Student’s t-test); *, *P* < 0.05; **, *P* < 0.01; ***, *P* < 0.001. **P** and **Q** Co-IP and IB analyses in HEK-293T cells transfected with the indicated constructs. IP was performed using an anti-Myc antibody, followed by IB with anti-Myc or anti-Flag antibodies. **R** Co-IP and IB assays in HEK-293T cells transfected with the indicated constructs. IP was performed with an anti-Myc antibody, followed by IB with anti-Flag, anti-Myc, or anti-HA antibodies. **S** and **T** Western blot analysis of Lyn degradation in Ctrl and *USP21*-KO lung cancer cells (H1299 in **S**, H460 in **T**) treated with cycloheximide at the indicated time points. Lyn degradation was quantified relative to GAPDH using ImageJ. Data are presented as mean ± SD from three independent experiments. Statistical significance (Student’s t-test); *, *P* < 0.05; **, *P* < 0.01; ***, *P* < 0.001. **U** Schematic model depicting the role of USP21 in stabilizing EGFR and Lyn. Ubiquitinated EGFR and Lyn are typically degraded via the lysosomal pathway. However, USP21 interacts with EGFR and Lyn to mediate their de-ubiquitination, leading to their stabilization. This stabilization enhances EGFR-mediated lung cancer progression

GSEA analysis further revealed that EGFR-associated signaling pathways were enriched in USP21^up^ patients (Fig. S5A-E). Patients exhibiting both USP21^up^ and EGFR^up^ expression (*n* = 26) showed significant enrichment of cancer-related and cell cycle-related gene sets compared to those with USP21^down^ and EGFR^down^ expression (*n* = 6) (Table S2, Fig. S6A-I, Fig. S6J-L). Lyn is known to regulate EGFR activation in lung cancer [10, 11], suggesting a functional association between USP21, EGFR, and Lyn in lung cancer. Notably, gene sets associated with cancer modules, lung cancer poor survival, cancer metastasis, and EGFR signaling were highly enriched in USP21^up^EGFR^up^Lyn^up^ patients (*n* = 4) as compared to those of USP21^down^EGFR^down^Lyn^down^ patients (*n* = 4) (Table S3; Fig. 1J; Fig. S7A-O).

Mechanistically, USP21 interacted directly with EGFR via its internal kinase domain (Fig. 1K-L, Fig. S8A-C) and promoted EGFR de-ubiquitination in a catalytic activity-dependent manner (Fig. 1M, Fig. S8D). Cycloheximide (CHX) chase assays revealed significantly reduced EGFR stability in *USP21*-KO cells compared to Ctrl cells (Fig. 1N-O). Additionally, Lyn interacted with EGFR (Fig. 1P). USP21 interacted with Lyn and promoted its de-ubiquitination (Fig. 1Q-R), leading to increased Lyn stability (Fig. 1S-T). These findings suggest that USP21 stabilizes both EGFR and Lyn, contributing to lung cancer progression (Fig. 1U).

To evaluate the role of USP21-EGFR-Lyn signaling in EGF-induced lung cancer progression, we examined downstream pathway activation in Ctrl and *USP21*-KO cells. Upon EGF stimulation, activation of EGFR, Lyn, AKT, IKKs, and p65 was significantly enhanced in Ctrl H1299 and Ctrl A549 cells but markedly reduced in *USP21*-KO cells (Fig. S9A-B). Functionally, *USP21*-KO cells exhibited reduced proliferation, migration, colony formation, and 3D spheroid formation in response to EGF stimulation (Fig. 2A-I, Fig. S10A-I). In rescue experiments, expression of Flag-USP21 in *USP21*-KO H1299 cells significantly restored EGF-induced spheroid formation (Fig. S11A-D). Additionally, overexpression of Flag-USP21 in control H1299 cells enhanced spheroid formation compared to mock-transfected controls (Fig. S11A-D). We further examined whether inhibition of USP21 using the small-molecule inhibitor BAY-805 [12] affects the expression of EGFR and Lyn, as well as EGF-induced cell proliferation and migration in EGFR-mutant lung cancer cell lines—H1975 (harboring the T790M EGFR mutation) and HCC827 (with an EGFR exon 19 deletion). Treatment with BAY-805 significantly reduced the expression of EGFR and Lyn not only in EGFR wild-type H1299 cells but also in the EGFR-mutant H1975 and HCC827 cells (Fig. S12A, H1299; Fig. S12B, H1975; Fig. S12C, HCC827). Furthermore, BAY-805 robustly inhibited cell proliferation and migration in H1299, H1975, and HCC827 cells (Fig. S13A-C, cell proliferation assay; Fig. S14A-B, migration assay), suggesting that USP21 regulates EGFR and Lyn expression in both EGFR wild-type and mutant lung cancer cells. Finally, we assessed the therapeutic potential of USP21 inhibition using 3D tumor spheroid models. The IC_50_ values of BAY-805 were determined for three lung cancer cell lines: 16.9 µM in H1299, 12.07 µM in H460, and 34.6 µM in A549 cells (Fig. S15A-F). Once spheroids stabilized, treatment with BAY-805 at its IC_50_ concentration significantly suppressed EGF-induced spheroid growth of A549, H460, and H1299 cells compared to untreated each control cell (Fig. 2J-M). Similar results could be observed in EGFR-mutant lung cancer cell lines, HCC827 (with an EGFR exon 19 deletion) and H1975 (harboring the T790M EGFR mutation), (Fig. S16A and B, HCC827; Fig. S16C and D, H1975). These findings suggest that USP21 inhibition may serve as a promising strategy to counteract EGF-induced tumor formation through the regulation of EGFR and Lyn expression (Fig. 2N).

**Fig. 2:**
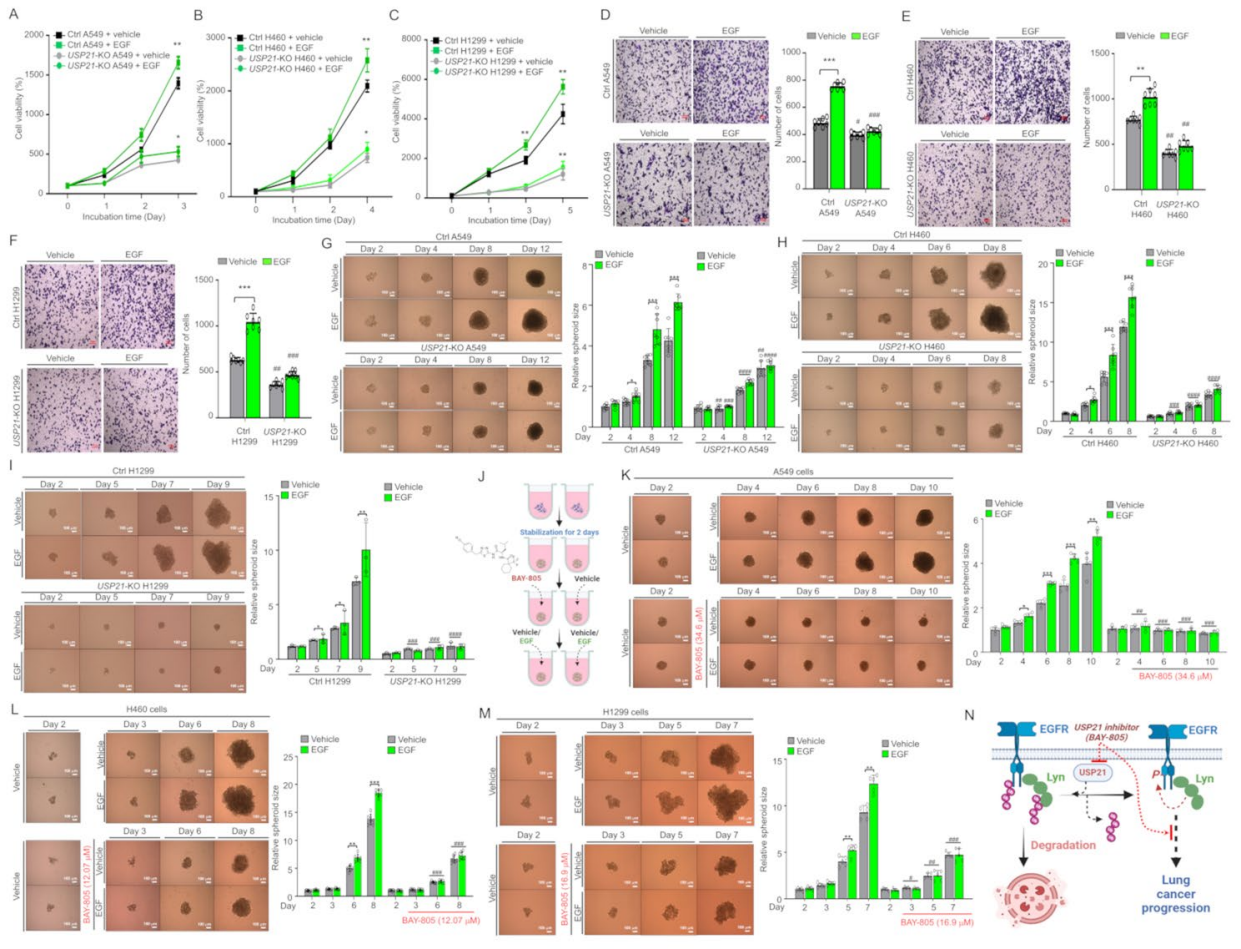
USP21 as a potential therapeutic target in EGF-induced lung cancer progression. **A-C** MTT assays were performed on control (Ctrl) and *USP21*-knockout (*USP21*-KO) lung cancer cells, including A549 (**A**), H460 (**B**), and H1299 (**C**) cells, treated with either vehicle or EGF. Results are presented as mean ± SD (*n* = 5). Statistical significance (Student’s t-test): *, *P* < 0.05; **, *P* < 0.01. **D-F** Transwell migration assays were conducted on Ctrl and *USP21*-KO cells, including A549 (**D**), H460 (**E**), and H1299 (**F**) cells, following vehicle or EGF treatment. Results are presented as mean ± SD (*n* = 7). Statistical significance (Student’s t-test): **, *P* < 0.01; ***, *P* < 0.001. ^#^, *P* < 0.05; ^##^, *P* < 0.01; ^###^, *P* < 0.001—comparisons between *USP21*-KO cells and their respective Ctrl cells. **G-I** 3D spheroid formation assays were performed using Ctrl and *USP21*-KO cells, including A549 (**G**), H460 (**H**), and H1299 (**I**), which were seeded in 96-well plates and incubated at 37 °C for 48 h to allow spheroid formation. Spheroids were then treated with either vehicle or EGF and cultured further as indicated. Spheroid size was measured using ImageJ software, and images were captured using phase-contrast microscopy (scale bar = 100 μm). Data are presented as mean ± SD (**G** and **H**, *n* = 7; **I**, *n* = 3). Statistical significance (Student’s t-test): *, *P* < 0.05; **, *P* < 0.01; ***, *P* < 0.001. ^#^, *P* < 0.05; ^##^, *P* < 0.01; ^###^, *P* < 0.001; ^####^, *P* < 0.0001—comparisons between *USP21*-KO cells and their respective Ctrl cells. **J** Schematic representation of the experimental protocol used to evaluate the inhibitory effects of BAY-805 on EGF-induced 3D tumor spheroid formation in lung cancer cells. Stabilized spheroids were incubated at 37 °C for 48 h, pre-treated with either vehicle or BAY-805 for 24 h, and then treated with either vehicle or EGF, followed by further culturing for different time points. **K-M** 3D tumor spheroid formation assays were conducted with A549 (**K**), H460 (**L**), and H1299 (**M**) cells. Spheroids were pre-treated with either vehicle or BAY-805 at specific concentrations (34.6 µM for A549 in **K**, 12.07 µM for H460 in **L**, and 16.9 µM for H1299 in **M**) for 24 h, followed by treatment with either vehicle or EGF. Spheroid size was measured using ImageJ software, and images were captured via phase-contrast microscopy (scale bar = 100 μm). Data are presented as mean ± SD (**K**, *n* = 5; **L**, *n* = 7; **M**, *n* = 5). Statistical significance (Student’s t-test): *, *P* < 0.05; **, *P* < 0.01; ***, *P* < 0.001. ^#^, *P* < 0.05; ^##^, *P* < 0.01; ^###^, *P* < 0.001—comparisons between cells treated with and without BAY-805. **N** Schematic model illustrating the role of USP21 in lung cancer progression through the USP21-EGFR-Lyn axis and its potential as a therapeutic target. Normally, ubiquitinated EGFR and Lyn undergo degradation via the lysosomal pathway. However, USP21 interacts with EGFR and Lyn, mediating their de-ubiquitination, leading to their stabilization and subsequent enhancement of EGFR-driven lung cancer progression. Inhibiting USP21 with compounds such as BAY-805 may provide a promising therapeutic strategy to counteract EGF-induced lung cancer progression

This study highlights the critical role of the USP21–EGFR–Lyn signaling axis in the progression of NSCLC. USP21, a deubiquitinase, was found to be significantly upregulated in NSCLC tumor tissues, correlating with gene signatures involved in tumor progression. Mechanistically, USP21 stabilizes both EGFR and Lyn, a Src family kinase, by preventing their ubiquitination and subsequent proteasomal degradation. This stabilization enhances downstream signaling pathways activated by EGF, leading to increased tumor cell proliferation, migration, and 3D spheroid formation in vitro -hallmarks of aggressive tumor behavior. Importantly, this study explores the therapeutic potential of BAY-805, a selective pharmacological inhibitor of USP21, in NSCLC. Treatment with BAY-805 led to decreased expression of EGFR and Lyn, significantly suppressing EGFR-driven oncogenic activity across various NSCLC cell lines, including A549 (EGFR wild-type and KRAS-mutant), H1299 (EGFR and KRAS wild-type), H460 (EGFR wild-type and KRAS-mutant), HCC827 (EGFR-mutant and KRAS wild-type), and H1975 (EGFR-mutant and KRAS wild-type). These results demonstrate the broad efficacy of USP21 inhibition across genetically diverse NSCLC subtypes. Considering the critical role of EGFR mutations and amplifications in prognosis and treatment decisions, the finding that USP21 regulates both wild-type and mutant EGFR expression provides compelling support for USP21-targeted therapeutic strategies. Overall, the data reveal a novel regulatory pathway involving USP21, EGFR, and Lyn, and highlight USP21 inhibition as a promising approach to modulate EGFR amplification and overexpression in NSCLC.

## Electronic supplementary material

Below is the link to the electronic supplementary material.

Supplementary Material 1

Supplementary Material 2

Supplementary Material 3

Supplementary Material 4

## Data Availability

No datasets were generated or analysed during the current study.

## Abbreviations

NSCLC: Non-small cell lung cancer
EGFR: Epidermal growth factor receptor
EGFR-TKIs: EGFR-tyrosine kinase inhibitors
USP21: Ubiquitin specific peptidase 21
Lyn: Lck/Yes novel tyrosine kinase
GSEA: Gene set enrichment analysis
CRISPR-Cas9: Clustered regularly interspaced short palindromic repeats and CRISPR-associated protein 9

## References

[1] Chen Z, Fillmore CM, Hammerman PS, Kim CF, Wong KK. Non-small-cell lung cancers: a heterogeneous set of diseases. Nat Rev Cancer. 2014;14(8):535–46. 25056707 10.1038/nrc3775 PMC5712844

[2] O’Leary C, Gasper H, Sahin KB, Tang M, Kulasinghe A, Adams MN, et al. Epidermal growth factor receptor (EGFR)-Mutated Non-Small-Cell lung Cancer (NSCLC). Pharmaceuticals (Basel). 2020;13(10):273. 32992872 10.3390/ph13100273 PMC7600164

[3] Huang L, Fu L. Mechanisms of resistance to EGFR tyrosine kinase inhibitors. Acta Pharm Sin B. 2015;5(5):390–401. 26579470 10.1016/j.apsb.2015.07.001 PMC4629442

[4] Koulouris A, Tsagkaris C, Corriero AC, Metro G, Mountzios G. Resistance to TKIs in EGFR-Mutated Non-Small cell lung cancer: from mechanisms to new therapeutic strategies. Cancers (Basel). 2022;14(14):3337. 35884398 10.3390/cancers14143337 PMC9320011

[5] Nukaga S, Yasuda H, Tsuchihara K, Hamamoto J, Masuzawa K, Kawada I, et al. Amplification of EGFR Wild-Type alleles in Non-Small cell lung Cancer cells confers acquired resistance to Mutation-Selective EGFR tyrosine kinase inhibitors. Cancer Res. 2017;77(8):2078–89. 28202511 10.1158/0008-5472.CAN-16-2359

[6] Kim JY, Shin JH, Kim MJ, Choi B, Kang Y, Choi J, et al. PTK2 is a potential biomarker and therapeutic target for EGFR- or TLRs-induced lung cancer progression via the regulation of the cross-talk between EGFR- and TLRs-mediated signals. Biomark Res. 2024;12(1):52. 38816856 10.1186/s40364-024-00604-x PMC11141017

[7] Kim MJ, Kim JY, Shin JH, Son J, Kang Y, Jeong SK, et al. The SARS-CoV-2 Spike protein induces lung cancer migration and invasion in a TLR2-dependent manner. Cancer Commun (Lond). 2024;44(2):273–7. 37702496 10.1002/cac2.12485 PMC10876188

[8] Shin JH, Kim MJ, Kim JY, Choi B, Kang Y, Kim SH, et al. USP21-EGFR signaling axis is functionally implicated in metastatic colorectal cancer. Cell Death Discov. 2024;10(1):492. 39695128 10.1038/s41420-024-02255-1 PMC11655878

[9] Shin JH, Kim MJ, Kim JY, Kang Y, Kim DH, Jeong SK, et al. CXCR5 and TLR4 signals synergistically enhance non-small cell lung cancer progression. Clin Transl Med. 2024;14(1):e1547. 38239075 10.1002/ctm2.1547 PMC10797246

[10] Sutton P, Borgia JA, Bonomi P, Plate JM. Lyn, a Src family kinase, regulates activation of epidermal growth factor receptors in lung adenocarcinoma cells. Mol Cancer. 2013;12:76. 23866081 10.1186/1476-4598-12-76 PMC3725175

[11] Sarıyar E, Karpat O, Sezan S, Baylan SM, Kıpçak A, Guven K, et al. EGFR and Lyn Inhibition augments regorafenib induced cell death in Sorafenib resistant 3D tumor spheroid model. Cell Signal. 2023;105:110608. 36693455 10.1016/j.cellsig.2023.110608

[12] Göricke F, Vu V, Smith L, Scheib U, Böhm R, Akkilic N, et al. Discovery and characterization of BAY-805, a potent and selective inhibitor of Ubiquitin-Specific protease USP21. J Med Chem. 2023;66(5):3431–47. 36802665 10.1021/acs.jmedchem.2c01933 PMC10009755

