# Supplementary material for: USP21-EGFR-Lyn axis drives NSCLC progression and therapeutic potential of USP21 inhibition
Source: Biomark Res. 2025 Jul 9;13:95. doi: 10.1186/s40364-025-00806-x (PMC12239452; doi:10.1186/s40364-025-00806-x)
Supplement: Supplementary file 4 — Supplementary Material 4 [file 40364_2025_806_MOESM4_ESM.docx]

**Additional Information**

**1. Supplementary Materials and Methods**

**2. Supplementary References**

**3. Supplementary Figures Legends**

**- Figure S1.** Gene enrichment analysis in NSCLC patients with differential USP21 expression

**- Figure S2.** Generation of *USP21*-Knockout (*USP21*-KO) lung cancer cells

**- Figure S3.** *In vitro* cancer progression assay with *USP21*-knockout (*USP21*-KO) lung cancer cells and control (Ctrl) cells

**- Figure S4.** 3D tumor spheroid formation assay in *USP21*-knockout (*USP21*-KO) and control (Ctrl) cells

**- Figure S5.** Gene enrichment analysis in NSCLC patients with differential USP21 expression

**- Figure S6.** Gene enrichment analysis in NSCLC patients with differential EGFR and USP21 expression

**- Figure S7.** Gene enrichment analysis in NSCLC patients with differential Lyn, EGFR, and USP21 expression

**- Figure S8.** De-ubiquitination assay with USP21 wild-type (WT) and USP21 C221A mutant vectors

**- Figure S9.** Western blotting analysis in control (Ctrl) and *USP21*-KO lung cancer cells in response to EGF

**- Figure S10.** *In vitro* cancer progression assay with *USP21*-knockout (*USP21*-KO) lung cancer cells and control (Ctrl) cells in response to EGF

**- Figure S11.** Rescue experiment in *USP21*-KO lung cancer cells transfected with USP21 vector

**- Figure S12.** Western blot analysis of EGFR and Lyn expression in EGFR wild-type (WT) and EGFR mutant lung cancer cells treated with BAY-805

**- Figure S13.** Cell proliferation assay in EGFR wild-type (WT) and EGFR mutant lung cancer cells treated with BAY-805

**- Figure S14.** Transwell migration assay in EGFR wild-type (WT) and EGFR mutant lung cancer cells treated with BAY-805

**- Figure S15.** Determination of IC_50_ concentration of BAY-805 on 3D tumor spheroid formation

**- Figure S16.** 3D tumor spheroid formation assays with H1975 (EGFR T790M mutant) and HCC827 (EGFR exon 19 deletion) treated with BAY-805

**- Figure S17.** Raw Western Blot data

**4. Supplementary Tables**

**- Table S1.** Clinical characteristics of NSCLC patients and ∆Mag (∆USP21) of USP21 in NSCLC patients (*n*=42)

**- Table S2.** Differential magnitude (∆Mag) of EGFR and USP21 expression in NSCLC patients (*n*=42)

**- Table S3.** Differential magnitude (∆Mag) of Lyn, EGFR, and USP21 expression in NSCLC patients (*n*=42)

**Supplementary Materials and methods**

**Patients and samples**

This study was conducted in accordance with the ethical principles stated in the Declaration of Helsinki. It was approved by the Institutional Review Board (IRB#: 2010-07-204) of Samsung Medical Center (SMC, Seoul, Korea). Written informed consent to use pathological specimens for research was obtained from all patients before surgery. Clinical data including gender, age, histology, cancer stage, and surgery were obtained from clinical records (Table S1). Lung tumor tissues and matched lung normal tissues of NSCLC patients (*n* = 42, Table S1) who had been diagnosed with lung cancer stage IA to stage IIIA were obtained from SMC. Lung tumor and matched normal specimens of enrolled patients were immediately frozen in liquid nitrogen and stored at −80 °C until use. Lung tumor tissues and matched lung normal tissues were verified by the Department of Laboratory Medicine and Pathology at SMC.

**Xenografted NSG mouse model**

NOD/SCID/IL-2Rγnull (NSG) mice were purchased from the Jackson Laboratory (Bar Harbor, ME, USA) and maintained under specific pathogen-free conditions in accordance with ethical guidelines for the care of these mice at the Bioanalysis Center Animal Facility, GenNBio Inc. (Seongnam, Korea). All experimental procedures were approved by the Institutional Animal Care and Use Committee (IACUC) of the Bioanalysis Center Animal Facility (IACUC #: 23-10-01). NSG mice at 6–8 weeks old were used to generate xenografted NSG mice. Control (Ctrl) H1299 (5 × 10^6^ cells per mouse, *n* = 5) or *USP21*-knockout (KO) H1299 cells (5 × 10^6^ cells per mouse, *n* = 5) in serum-free RPMI were injected under NSG mice skin (back area) [1-3]. The final injection volume was 100 μL/mouse containing a 1:1 v/v mixture of ice-chilled Matrigel (BD Biosciences, La Jolla, CA, USA), which was kept on ice until injection. After injecting cancer cells, tumor volume was measured with a caliper until 58 days after injection. Tumor volumes (mm^3^) were calculated as (length x width^2^) × 0.5. Tumor growth curves are presented as average tumor volume ± SEM for each group in this study. All studies involving mice were approved by the Nemours IACUC.

**Cells**

A549 cells (human lung cancer cell line; CCL-185, American Type Culture Collection (ATCC), Manassas, VA, USA), H1299 cells (human non-small cell lung cancer cell line; CRL-5803, ATCC), H460 cells (human large cell lung cancer cell line; HTB-177, ATCC), H1975 cells (human lung cancer cell line harboring the T790M EGFR mutation; CRL-5908, ATCC), and HCC827 cells (human non-small cell lung cancer (NSCLC) cell line carrying an EGFR exon 19 deletion; KCLB No. 70827, Korean Cell Line Bank (KCLB), Seoul, Korea) were maintained in RPMI 1640 medium (LM011-01, Welgene, Daegu, Korea) supplemented with 10% fetal bovine serum (FBS), penicillin (100 μg/mL), and streptomycin (100 μg/mL) in a 5% CO_2_ humidified atmosphere at 37 °C. Human embryonic kidney (HEK) 293T cells (CRL-11268, ATCC) were cultured and maintained in Dulbecco's modified Eagle's medium (DMEM; LM001-05, Welgene, Daegu, Korea) with 10% FBS.

**Antibodies and reagents**

Anti-Myc (sc40), anti-USP21 (sc-515911), anti-HA (sc-7392), anti-phospho-AKT1 (sc-52940), and anti-GAPDH (sc-47724) antibodies were purchased from Santa Cruz Biotechnology (Santa Cruz, CA, USA). Anti-Flag (F3165) antibody was purchased from Sigma-Aldrich (St. Louis, MO, USA). Anti-EGFR (2232S), anti-phospho-EGFR (2236S), anti-Lyn (2796S), anti-phospho-Lyn (Tyr507) (2731S), anti-IKKβ (2684S), anti-phospho-IKKα/β (Ser176/180) (2697S), anti-NF-kappaB p65 (8242S), anti-p-NF-kappaB p65 (3033S), and anti-AKT1 (75692) antibodies were purchased from Cell Signaling Technology (Danvers, MA, USA). TrueBlot® secondary antibodies (18-8816-33, 18-8817-33) were purchased from Rockland Immunochemicals (Pottstown, PA, USA). Goat anti-rabbit IgG (HRP) (GTX213110-01) antibody was purchased from GeneTex Inc. (Irvine, CA, USA). Rabbit anti-mouse IgG H&L (HRP) (ab6728) antibody was purchased from Abcam (Cambridge, MA, USA). Dimethyl sulfoxide (DMSO; D4540), phosphate-buffered saline (PBS; CBP007A), glutaraldehyde (G6257-100ml), crystal violet (C6158-50g), cycloheximide (CHX; C1988), EGF (SRP3027), and thiazolyl blue tetrazolium bromide (MTT; M5655) were purchased from Sigma-Aldrich (St. Louis, MO, USA). Lipofectamine 2000 (11668019) and Opti-MEM (31985070) were purchased from Thermo Fisher Scientific (Waltham, MA, USA). Agarose powder (AGA001.500) was purchased from Bioshop Canada (Burlington, ON, CAN). Protein G beads (17-0618-02) were purchased from GE healthcare (Chicago, IL, USA). Transwell® with 8.0 µm Pore Polycarbonate Membrane Insert (3422) was purchased from Corning (NY, USA). BAY-805 (HY-153045) was purchased from MedChemExpress (Monmouth Junction, NJ, USA).

**Plasmid constructs**

EGFR WT (11011), HA-Ubiquitin (18712), and Flag-HA-USP21 (22574) were purchased from Addgene (Watertown, MA, USA). pCMV-3Tag-7 (240202) and pCMV-3Tag-6 (240200) were purchased from Agilent Technologies (Santa Clara, CA, USA). The full-length of Lyn was generated by PCR using cDNA library as a template and inserted into the pCMV-3Tag-7 or pCMV-3Tag-6 vector to generate Flag- or Myc-Lyn vectors. The full-length of EGFR and USP21 were cloned into pCMV-3Tag-7 or pCMV-3Tag-6 vectors to generate various Flag- or Myc-tagged constructs. Truncated mutants of Flag-EGFR (Flag-EGFR 669-1210 and Flag-EGFR 954-1210) were generated by PCR using Flag-EGFR WT plasmid as a template and inserted into pCMV-3Tag-6 vector. Flag-USP21 C221A mutant was generated by site-directed mutagenesis using Flag-USP21 WT plasmid.

**Generation of *USP21*-Knockout (*USP21*-KO) cell lines with CRISPR/Cas9**

To generate *USP21*-KO lung cancer cells with CRISPR/Cas9 gene editing method, we used two vector systems, including single guide RNA (sgRNA) and CRISPR-associated protein 9 (Cas9) vectors. sgRNA and Cas9 vectors were kindly provided by Dr. Daesik Kim (Sungkyunkwan University School of Medicine, Suwon, Korea). Guide RNA sequences for CRISPR/Cas9 were designed on the CRISPR design website (http://crispr.mit.edu/) provided by the Feng Zhang Lab. Insert oligonucleotides for human USP21 gRNA were: 5’-TCATGTTGGCCTTCGAAACC-3’ (gRNA-1) / 5’-AGTAGGATTCACAGCTTCGC-3’ (gRNA-2) / 5’-CTTCTCTGGATACAGCCAGC-3’ (gRNA-3). Complementary oligonucleotides to guide RNAs (gRNAs) were annealed and cloned into a sgRNA vector. sgRNA vectors expressing gRNA of USP21, and Cas9 vector expressing Cas9 were transfected into A549, H1299, and H460 lung cancer cells using Lipofectamine 2000 (Thermo Fisher Scientific, Waltham, MA, USA) according to the manufacturer’s instructions. After two weeks, colonies were isolated from 96-well plates and expression levels of USP21 were analyzed with western blotting.

**Immunoprecipitation (IP) assay**

HEK-293T cells were transiently transfected with mock (a relevant control vector), Flag-EGFR, Flag-EGFR 669-1210, Flag-EGFR 954-1210, Myc-EGFR, Flag-Lyn, Flag-USP21, or Myc-USP21 as indicated in each figure for 24 hr. After collecting cells, cell lysates were prepared and immunoprecipitated with anti-Myc or anti-Flag antibodies as indicated. IP complexes were separated by sodium dodecyl sulfate-polyacrylamide gel electrophoresis (SDS-PAGE, 8–12%) and immune-probed with anti-Myc or anti-Flag antibodies.

**Ubiquitination assay**

HEK-293T cells were transiently transfected with mock, HA-Ub, Myc-EGFR, Myc-Lyn, Flag-USP21, or Flag-USP21 C221A as indicated in each figure for 24 hr. After collecting cells, cell lysates were prepared and immunoprecipitated with anti-Myc antibody. IP complexes were separated by sodium dodecyl sulfate-polyacrylamide gel electrophoresis (SDS-PAGE, 8–12%) and immune-probed with anti-HA, anti-Myc, or anti-Flag antibodies.

**Western Blotting (WB) assay**

Control (Ctrl) A549, Ctrl H1299, *USP21*-KO A549, and *USP21*-KO H1299 cells were seeded into 6-well plates and cultured. Cells were stimulated with vehicle (DMSO, 0.1% v/v) or EGF (5 ng/mL) for 30 min. After collecting cells, cell lysates were separated by sodium dodecyl sulfate-polyacrylamide gel electrophoresis (SDS-PAGE, 8–12 %) and immune-probed with anti-EGFR, anti-phospho-EGFR, anti-Lyn, anti-phospho-Lyn, anti-phospho-IKKs, anti-IKKs, anti-p65, anti-phospho-p65, anti-phospho-AKT1, anti-AKT1, or anti-GAPDH (as loading control) antibodies. H1299 (EGFR wild-type), H1975 (EGFR T790M mutant), and HCC827 (EGFR exon 19 deletion) lung cancer cells were seeded into 12-well plates and cultured. Cells were stimulated with vehicle (DMSO, 0.1% v/v concentration) or different concentrations of BAY-805 (a USP21 inhibitor) for 12 hours. After collecting cells, cell lysates were separated by SDS-PAGE (8 -12%) and immune-probed with anti-EGFR, anti-Lyn, or anti-GAPDH (as loading control) antibodies.

**Cycloheximide (CHX) chase assay**

Cycloheximide (CHX) chase assay was performed to determine the half-life of EGFR or Lyn following previous protocols [4]. Briefly, Ctrl H460, Ctrl H1299, *USP21*-KO H460, and *USP21*-KO H1299 cells were treated with vehicle (DMSO, 0.1% v/v) or CHX (50 µg/mL; Sigma-Aldrich, St. Louis, MO, USA) for different time periods. The expression of EGFR or Lyn level was detected by western blotting assay with anti-EGFR, anti-Lyn, or anti-GAPDH (as loading control) antibodies.

**Transwell migration assay**

Transwell migration assay was performed as previously described [2,3,5,6]. Briefly, Ctrl A549, Ctrl H460, Ctrl H1299, *USP21*-KO A549, *USP21*-KO H460, and *USP21*-KO H1299 cells were suspended in 250 μL of culture medium and added to the upper compartment of a 24-well Transwell® chamber (8 μm pore; Corning, 3422). The cells were treated with vehicle (DMSO, 0.1% v/v) or EGF (10 ng/mL) and incubated at 37 °C for 24 hr. Migratory cells passed through the polycarbonate membrane and adhered to its bottom side, while non-migratory cells remained in the upper chamber. After non-migratory cells were removed, migratory cells were fixed with 2.5% glutaraldehyde (Sigma-Aldrich, G6257) and stained with 0.1% crystal violet (Sigma-Aldrich, C6158-50g). H1299 (EGFR wild-type), H1975 (EGFR T790M mutant), and HCC827 (EGFR exon 19 deletion) lung cancer cell lines were treated with either vehicle (0.1% DMSO) or BAY-805 (17 μM) for 24 hours. After incubation, cells were suspended in 250 μL of culture medium and added to the upper compartment of a 24-well Transwell® chamber (8 μm pore; Corning, 3422). The cells were treated with vehicle (DMSO, 0.1% v/v) or EGF (10 ng/mL) and incubated at 37 °C for 24 hr. Transwell migration assay was performed.

**Wound-healing migration assay**

Wound-healing migration assay was performed as following protocols [2,3,5,6]. Briefly, Ctrl A549, Ctrl H460, Ctrl H1299, *USP21*-KO A549, *USP21*-KO H460, and *USP21*-KO H1299 cells were seeded into 12-well plates and cultured to reach confluence. Cell monolayers were gently scratched, washed with culture medium to remove floating cells and debris, and treated with vehicle (DMSO, 0.1% v/v) or EGF (15–20 ng/mL) for various time periods. Images of cell migration were captured at indicated time points.

**Anchorage-independent soft agar colony formation assay**

Anchorage-independent soft agar colony formation assay was performed as following protocols [2,3]. Briefly, Ctrl A549, Ctrl H460, Ctrl H1299, *USP21*-KO A549, *USP21*-KO H460, and *USP21*-KO H1299 cells were mixed with 0.3% agarose (Biotechnology Grade, GA001.500) in complete medium and plated onto a bottom layer of 0.5% agarose in 24-well plates. Growth medium containing either vehicle (DMSO, 0.1% v/v) or EGF (10 ng/mL) was added on top, and the cells were incubated at 37 °C for 30 days.

**Anchorage-dependent colony formation assay**

Anchorage-dependent colony formation assay was performed as following protocols [2,3]. Briefly, Ctrl A549, Ctrl H460, Ctrl H1299, *USP21*-KO A549, *USP21*-KO H460, and *USP21*-KO H1299 cells were seeded into 12-well culture plates at a density of 300 cells/well and treated with vehicle (DMSO, 0.1% v/v) or EGF (10 ng/mL). After 9 days of incubation, colonies were fixed and stained with 0.5% crystal violet (Sigma-Aldrich, C6158-50g). The number of colonies was quantified using ImageJ software.

**MTT assay**

MTT assay was performed as following protocols [2,3]. Briefly, Ctrl A549, Ctrl H460, Ctrl H1299, *USP21*-KO A549, *USP21*-KO H460, and *USP21*-KO H1299 cells were seeded into 96-well culture plates at a density of 700 cells/well, treated with vehicle (DMSO, 0.1% v/v) or EGF (5 ng/mL), and incubated for various time periods. Cell viability was assessed using an MTT reagent (1 mg/mL in PBS; Sigma-Aldrich, M5655). On the measurement day, the medium was carefully replaced with 10% MTT and incubated at 37 °C for 3 hr. After incubation, formazan crystals were dissolved in 100 μL of DMSO, and MTT reduction was quantified by measuring absorbance at 595 nm using a Bio-Rad Model 680 microplate reader (Bio-Rad, CA, USA). H1299 (EGFR wild-type), H1975 (EGFR T790M mutant), and HCC827 (EGFR exon 19 deletion) lung cancer cell lines were seeded into 96-well plates at a density of 300 cells per well. Cells were treated with either vehicle (0.1% DMSO) or BAY-805 (17 μM) for 24 hours, followed by treatment with or without EGF (10 ng/mL). Cell viability was measured at the indicated time points using the MTT assay.

**Three-dimension (3D) spheroids formation assay using agarose-coated plates**

The 3D spheroids formation assay was performed following protocol [2,3,5]. Briefly, 1.5% agarose hydrogel was added to each well of 96-well culture plates and incubated at room temperature (RT) for 30 min. Ctrl A549, Ctrl H460, Ctrl H1299, *USP21*-KO A549, *USP21*-KO H460, and *USP21*-KO H1299 cells were seeded into 100 µl growth medium at a density of 50, 125, or 500 cells/well. The plates were incubated at 37 °C for 48 hours to allow spheroids formation. Spheroids were then treated with vehicle (DMSO, 0.1% v/v) or EGF (10 ng/mL) and incubated for additional time periods. Spheroid formation and growth were assessed using phase-contrast microscopy, and spheroid size was measured using ImageJ Software (National Institutes of Health, Bethesda, MD, USA). For the determination of IC_50_ value of BAY-805 in A549, H460, or H1299 spheroids, A549, H460, or H1299 lung cancer cells were seeded in 96-well plates at a density of 125 or 500 cells/well and incubated at 37 °C for 48 hours to allow spheroids formation. Spheroids were then treated with vehicle (DMSO, 0.1% v/v) or varying concentrations of BAY-805 and incubated for different time periods. The IC_50_ value of BAY-805 was calculated using GraphPad Prism 8.0 software. To evaluate the inhibitory effect of BAY-805 on EGF-induced spheroids in A549, H460, or H1299 cells, wild-type (WT) A549, H460, or H1299 lung cancer cells were seeded in 96-well plates at a density of 125 or 500 cells/well and incubated at 37 °C for 48 hours to allow spheroids formation. Spheroids were treated with vehicle (DMSO, 0.1% v/v) or BAY-805 at 34.6 μM for A549 spheroids, 12.07 μM for H460 spheroids, and 16.9 μM for H1299 spheroids. After 24 hours, spheroids were further treated with vehicle (DMSO, 0.1% v/v) or EGF (20 ng/mL). H1975 (EGFR T790M mutant) and HCC827 (EGFR exon 19 deletion) cells were seeded into 100 µl growth medium at a density of 250 cells/well. The plates were incubated at 37 °C for 48 hours to allow spheroids formation. Spheroids were pre-treated with either vehicle or 17 µM BAY-805 for 24 hours, followed by treatment with either vehicle or EGF (15 ng/ml). Tumor spheroid formation and growth were evaluated using phase-contrast microscopy, and spheroid sizes were measured using ImageJ software. (NIH, Bethesda, MD, USA).

**Rescue experiment**

Ctrl H1299 and *USP21*-KO H1299 cells transfected without, or with mock (a control vector) and Flag-USP21 vector were seeded in 96-well plates and incubated at 37°C for 48 hours to allow spheroid formation. Tumor spheroid formation assay was performed for different times. Spheroid size was measured using ImageJ software, and images were captured via phase-contrast microscopy.

**Microarray Analysis**

Microarray analysis was performed as previously described [7-9]. Total RNAs were extracted from tumor and matched normal tissues of 42 patients with NSCLC with Trizol (Thermo Fisher Scientific, 15596026) and purified using RNeasy columns (Qiagen, 74106) according to each manufacturer’s protocol.

**Gene Set Enrichment Analysis (GSEA)**

Different magnitudes (∆ Mags) of USP21, Lyn, and EGFR expression were obtained from microarray data between lung tumor tissues and matched lung normal tissues. Patients (*n* = 32) with up-regulated USP21 and patients (*n* = 10) with down-regulated USP21, patients (*n* = 26) with up-regulated EGFR and USP21 and patients (*n* = 6) with down-regulated EGFR and USP21, or patients (*n* = 4) with up-regulated Lyn, EGFR, and USP21 and patients (*n* = 4) with down-regulated Lyn, EGFR, and USP21 were selected based on ∆ Mags of these genes. To identify genes showing significant differences, normalized enrichment score (NES), nominal *P*-value, and FDR-q value were analyzed by GSEA (http://www.gsea-msigdb.org/gsea/index.jsp).

**Statistical Analysis**

All data are expressed as mean ± SD (standard deviation). Statistical significance was determined by Student’s t-test using GraphPad Prism 5.0 (GraphPad Software, San Diego, CA, USA). *P*-values are marked by asterisks (*, *P* < 0.05; **, *P* < 0.01; ***, *P* < 0.001; ****, *P* < 0.0001: ^#^, *P* < 0.05; ^##^, *P* < 0.01; ^###^, *P* < 0.001; ^####^, *P* < 0.0001).

**Supplementary References**

1. Kim MJ, Choi B, Kim JY, Min Y, Kwon DH, Son J, et al. USP8 regulates liver cancer progression via the inhibition of TRAF6-mediated signal for NF-κB activation and autophagy induction by TLR4. Transl Oncol. 2022;15(1):101250.

2. Kim JY, Shin JH, Kim MJ, Choi B, Kang Y, Choi J, et al. PTK2 is a potential biomarker and therapeutic target for EGFR- or TLRs-induced lung cancer progression via the regulation of the cross-talk between EGFR- and TLRs-mediated signals. Biomark Res. 2024;12(1):52.

3. Shin JH, Kim MJ, Kim JY, Choi B, Kang Y, Kim SH, et al. USP21-EGFR signaling axis is functionally implicated in metastatic colorectal cancer. Cell Death Discov. 2024;10(1):492.

4. Miao Y, Du Q, Zhang HG, Yuan Y, Zuo Y, Zheng H. Cycloheximide (CHX) Chase Assay to Examine Protein Half-life. Bio Protoc. 2023;13(11):e4690.

5. Shin JH, Kim MJ, Kim JY, Kang Y, Kim DH, Jeong SK, et al. CXCR5 and TLR4 signals synergistically enhance non-small cell lung cancer progression. Clin Transl Med. 2024;14(1):e1547.

6. Kim MJ, Kim JY, Shin JH, Son J, Kang Y, Jeong SK, et al. The SARS-CoV-2 spike protein induces lung cancer migration and invasion in a TLR2-dependent manner. Cancer Commun (Lond). 2024;44(2):273-277.

7. Kim MJ, Min Y, Son J, Kim JY, Lee JS, Kim DH, et al. AMPKα1 Regulates Lung and Breast Cancer Progression by Regulating TLR4-Mediated TRAF6-BECN1 Signaling Axis. Cancers (Basel). 2020;12(11):3289.

8. Min Y, Wi SM, Kang JA, Yang T, Park CS, Park SG, et al. Cereblon negatively regulates TLR4 signaling through the attenuation of ubiquitination of TRAF6. Cell Death Dis. 2016;7(7):e2313.

9. Kim Y, Lee BB, Kim D, Um S, Cho EY, Han J, et al. Clinicopathological Significance of RUNX1 in Non-Small Cell Lung Cancer. J Clin Med. 2020;9(6):1694.

**Supplementary Figure Legends**

**
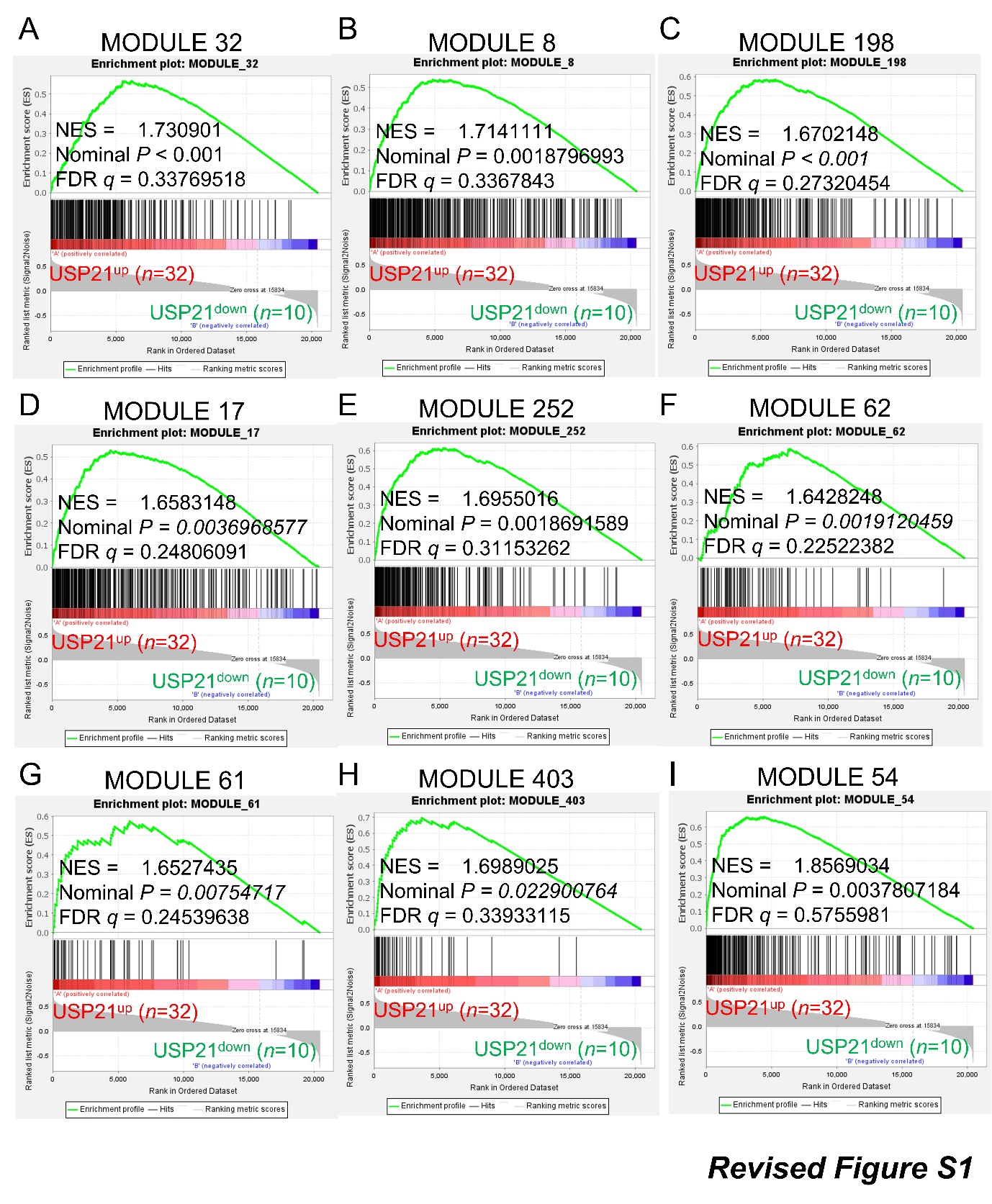
**

**Supplementary Figure S1.** Gene enrichment analysis in NSCLC patients with differential USP21 expression. **A*-*I** Using microarray data from lung tumor tissues (LTTs, *n* = 42) and matched lung normal tissues (mLNTs, *n* = 42), Δ Mag values for USP21 expression (AVG signal) were calculated and ranked. Patients were categorized based on USP21 expression levels; USP21-upregulated (USP21^up^) NSCLC patients (*n* = 32) and USP21-downregulated (USP21^down^) NSCLC patients (*n* = 10) (Fig. 1B, Table S1). GSEA was conducted comparing USP21^up^ (*n* = 32) and USP21^down^ (*n* = 10) NSCLC patients. Enriched cancer modules include: **A**, MODULE 32; **B**, MODULE 8; **C**, MODULE 198; **D**, MODULE 17; **E**, MODULE 252; **F**, MODULE 62; **G**, MODULE 61; **H**, MODULE 403; **I**, MODULE 54. Each panel displays the Normalized Enrichment Score (NES), nominal *p*-values, and False Discovery Rate (FDR) q-value.

**
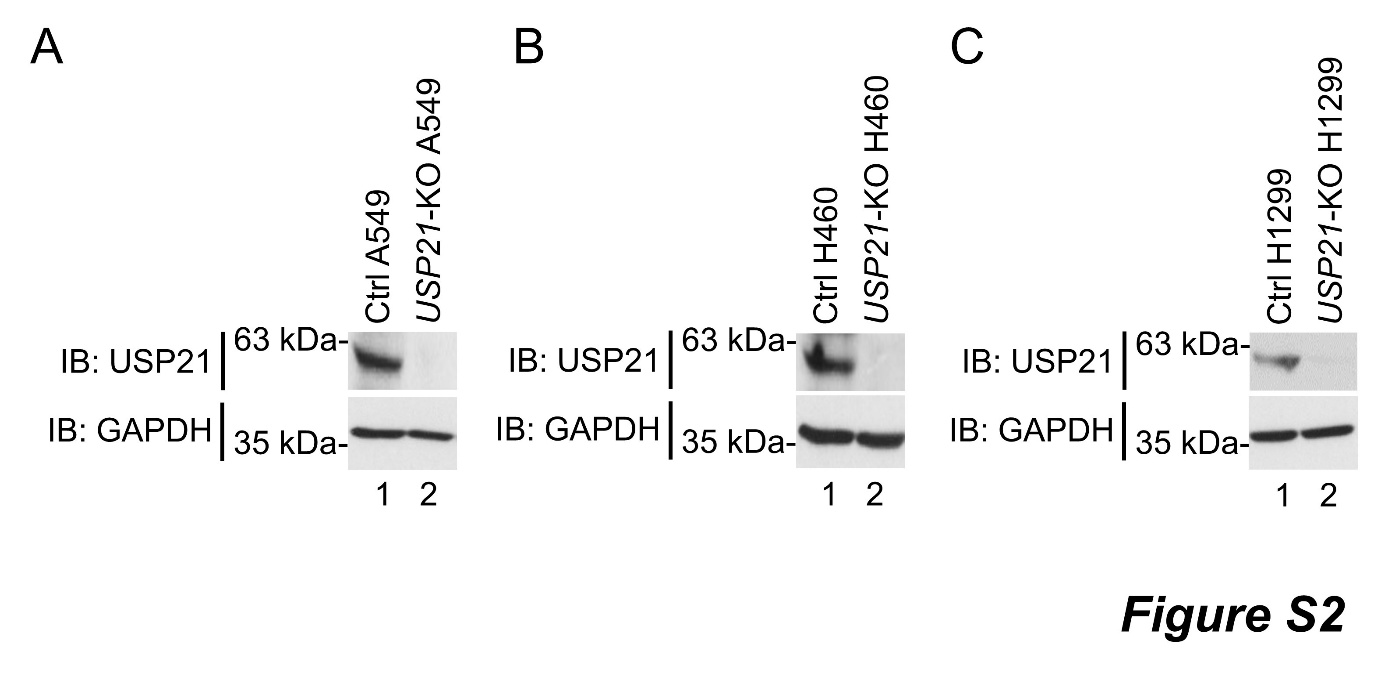
Supplementary Figure S2.** Generation of *USP21*-Knockout (*USP21*-KO) lung cancer cells. **A*-*C** sgRNA vectors expressing gRNA of USP21, and Cas9 vector expressing Cas9 were transfected into A549 (**A**), H460 (**B**), and H1299 (**C**) lung cancer cells. After two weeks, colonies were isolated from 96-well plates and expression levels of USP21 were analyzed with western blotting.

**
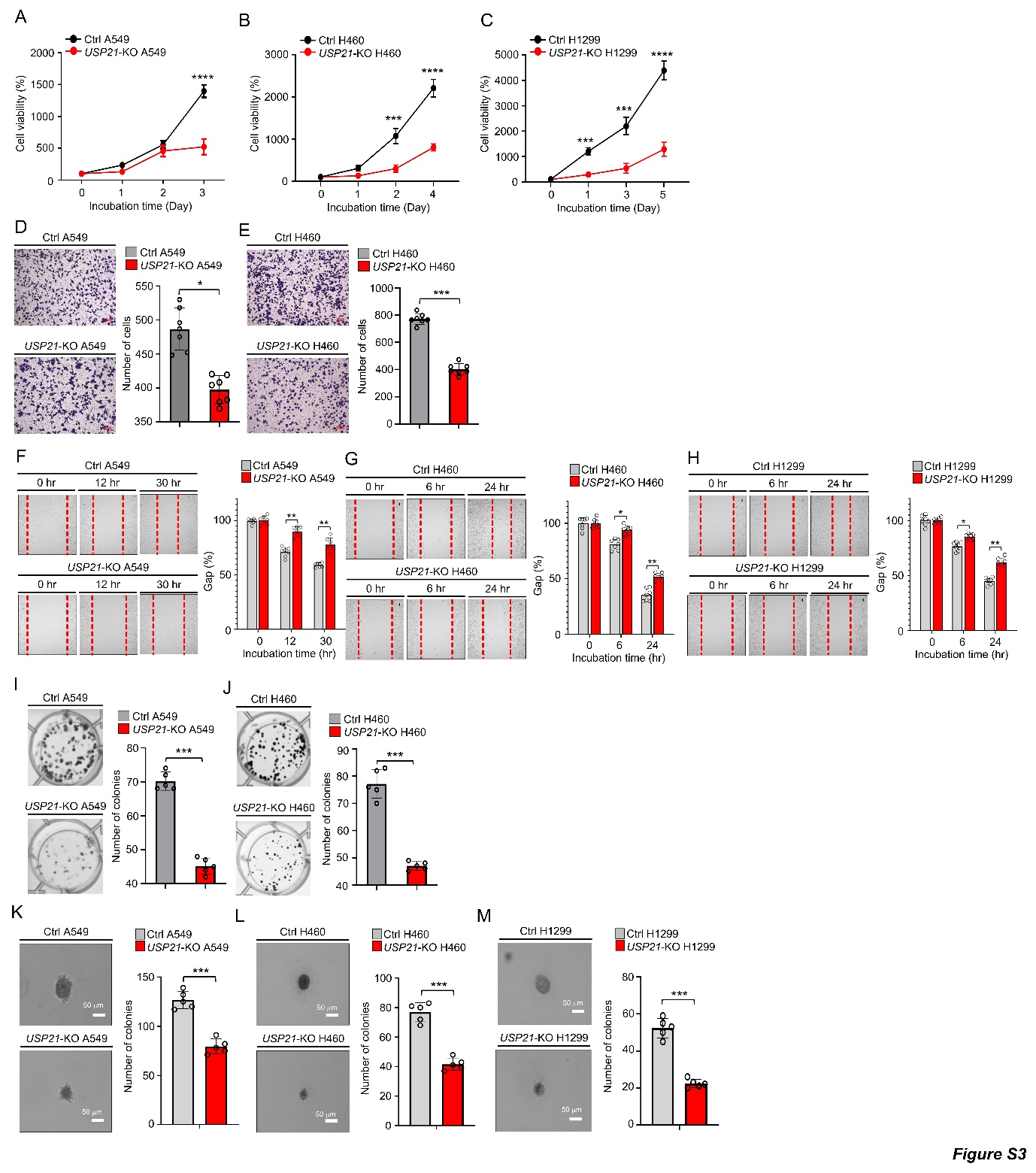
**

**Supplementary Figure S3.** *In vitro* cancer progression assay with *USP21*-knockout (*USP21*-KO) lung cancer cells and control (Ctrl) cells. **A*-*C** MTT assays were conducted on Ctrl A549 and *USP21*-KO A549 (**A**), Ctrl H460 and *USP21*-KO H460 (**B**), or Ctrl H1299 and *USP21*-KO H1299 cells (**C**). Results are presented as mean ± SD (*n* = 5). Statistical significance (Student’s t-test): ***, *P* < 0.001; ****, *P* < 0.0001. **D and E** Transwell migration assays were conducted on Ctrl A549 and *USP21*-KO A549 cells (**D**), Ctrl H460 and *USP21*-KO H460 cells (**E**). Results are presented as mean ± SD (*n* = 7). Statistical significance (Student’s t-test): *, *P* < 0.05; ***, *P* < 0.001. **F-H** Wound healing assays were conducted on Ctrl A549 and *USP21*-KO A549 (**F**), Ctrl H460 and *USP21*-KO H460 (**G**), or Ctrl H1299 and *USP21*-KO H1299 cells (**H**). Results are presented as mean ± SD (*n* = 7). Statistical significance (Student’s t-test): *, *P* < 0.05; **, *P* < 0.01. **I and J** Anchorage-dependent colony formation assays were conducted on Ctrl A549 and *USP21*-KO A549 (**I**) or Ctrl H460 and *USP21*-KO H460 cells (**J**). Results are presented as mean ± SD (*n* = 7). Statistical significance (Student’s t-test): ***, *P* < 0.001. **K-M** Anchorage-independent colony formation assays were conducted on Ctrl A549 and *USP21*-KO A549 (**K**), Ctrl H460 and *USP21*-KO H460 (**L**), or Ctrl H1299 and *USP21*-KO H1299 cells (**M**). Results are presented as mean ± SD (*n* = 7). Statistical significance (Student’s t-test): ***, *P* < 0.001.

**
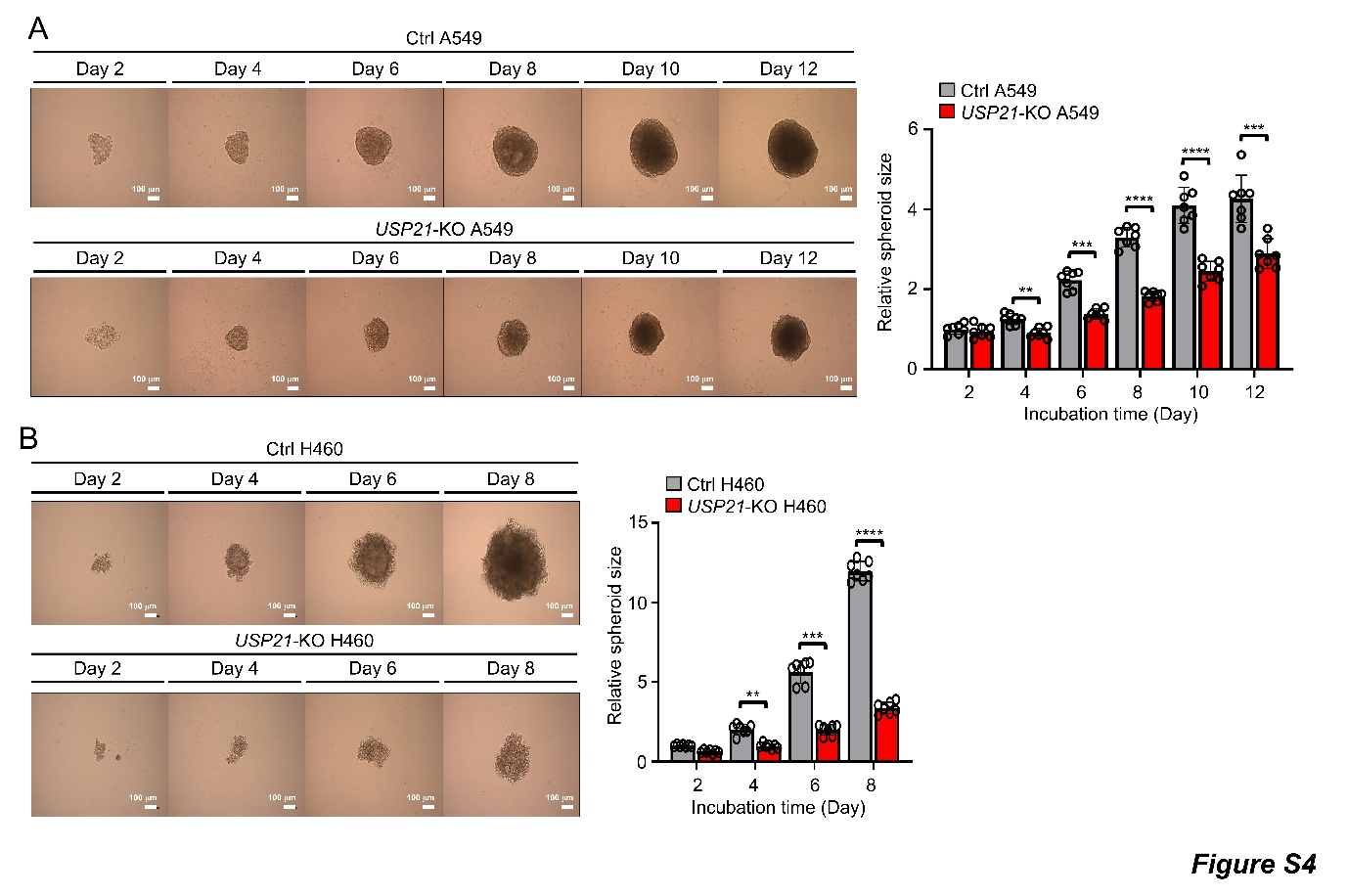
Supplementary Figure S4.** 3D tumor spheroid formation assay in *USP21*-knockout (*USP21*-KO) and control (Ctrl) cells. **A and B** 3D spheroid formation assays were performed using Ctrl A549 and *USP21*-KO A549 cells (**A**) or Ctrl H460 and *USP21*-KO H460 cells (**B**), which were seeded in 96-well plates and incubated at 37°C for 48 hours to allow spheroid formation. Spheroids were further incubated at different times, as indicated. Spheroid size was measured using ImageJ software, and images were captured using phase-contrast microscopy (scale bar = 100 µm). Data are presented as mean ± SD (*n* = 7). Statistical significance (Student’s t-test): **, *P* < 0.01; ***, *P* < 0.001; ****, *P* < 0.0001.

**
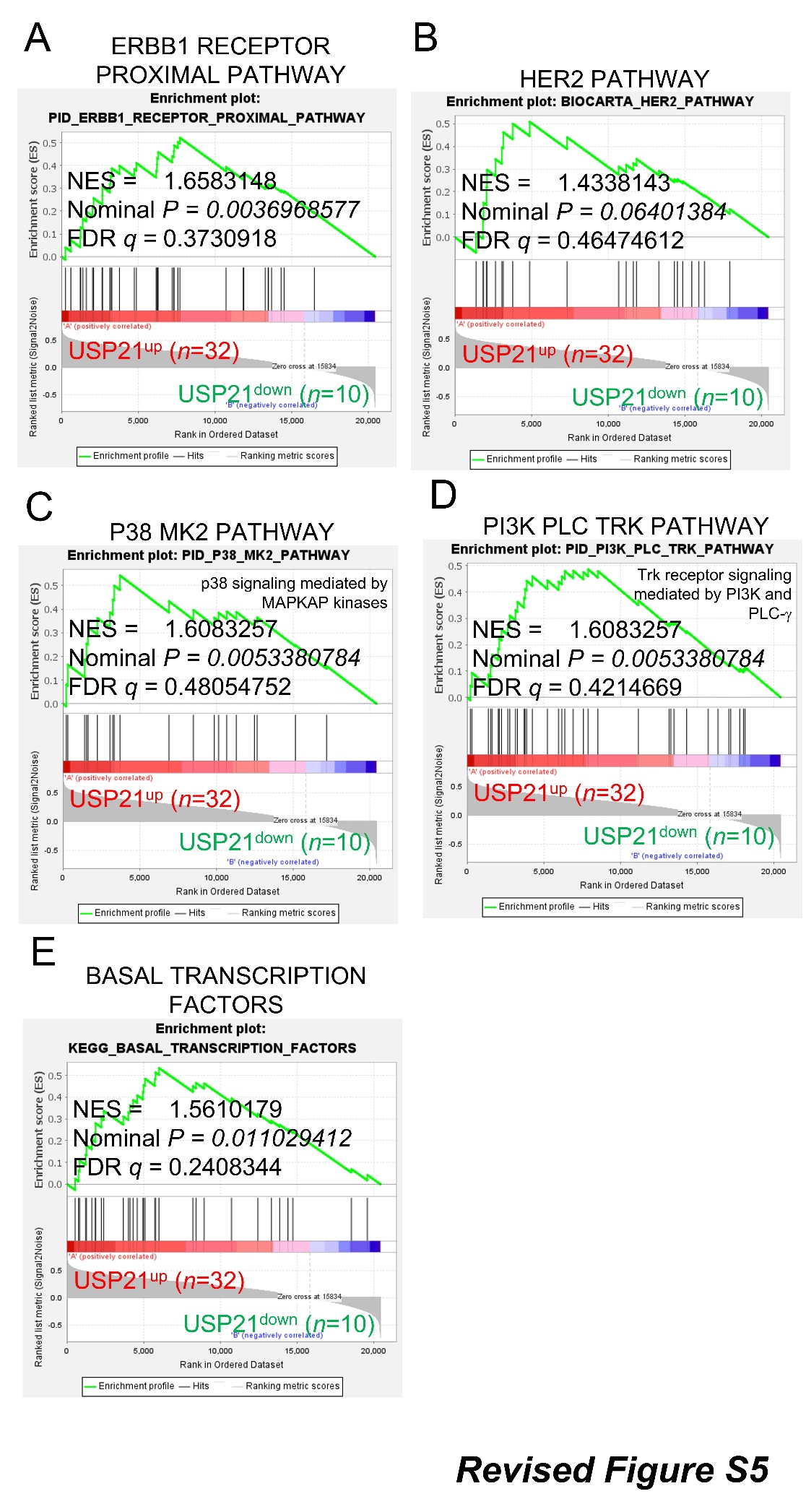
**

**Supplementary Figure S5.** Gene enrichment analysis in NSCLC patients with differential USP21 expression. **A*-*E** Using microarray data from lung tumor tissues (LTTs, *n* = 42) and matched lung normal tissues (mLNTs, *n* = 42), Δ Mag values for USP21 expression (AVG signal) were calculated and ranked. Patients were categorized based on USP21 expression levels; USP21-upregulated (USP21^up^) NSCLC patients (*n* = 32) and USP21-downregulated (USP21^down^) NSCLC patients (*n* = 10) (Fig. 1B, Table S1). GSEA was conducted comparing USP21^up^ (*n* = 32) and USP21^down^ (*n* = 10) NSCLC patients. Enriched EGFR-associated pathways include: **A**, ERBB1 RECEPTOR PROXIMAL PATHWAY; **B**, HER2 PATHWAY; **C**, P38 MK2 PATHWAY; **D**, PI3K PLC TRK PATHWAY; **E**, BASAL TRANSCRIPTION FACTORS. Each panel displays the Normalized Enrichment Score (NES), nominal *p*-values, and False Discovery Rate (FDR) q-value.

**
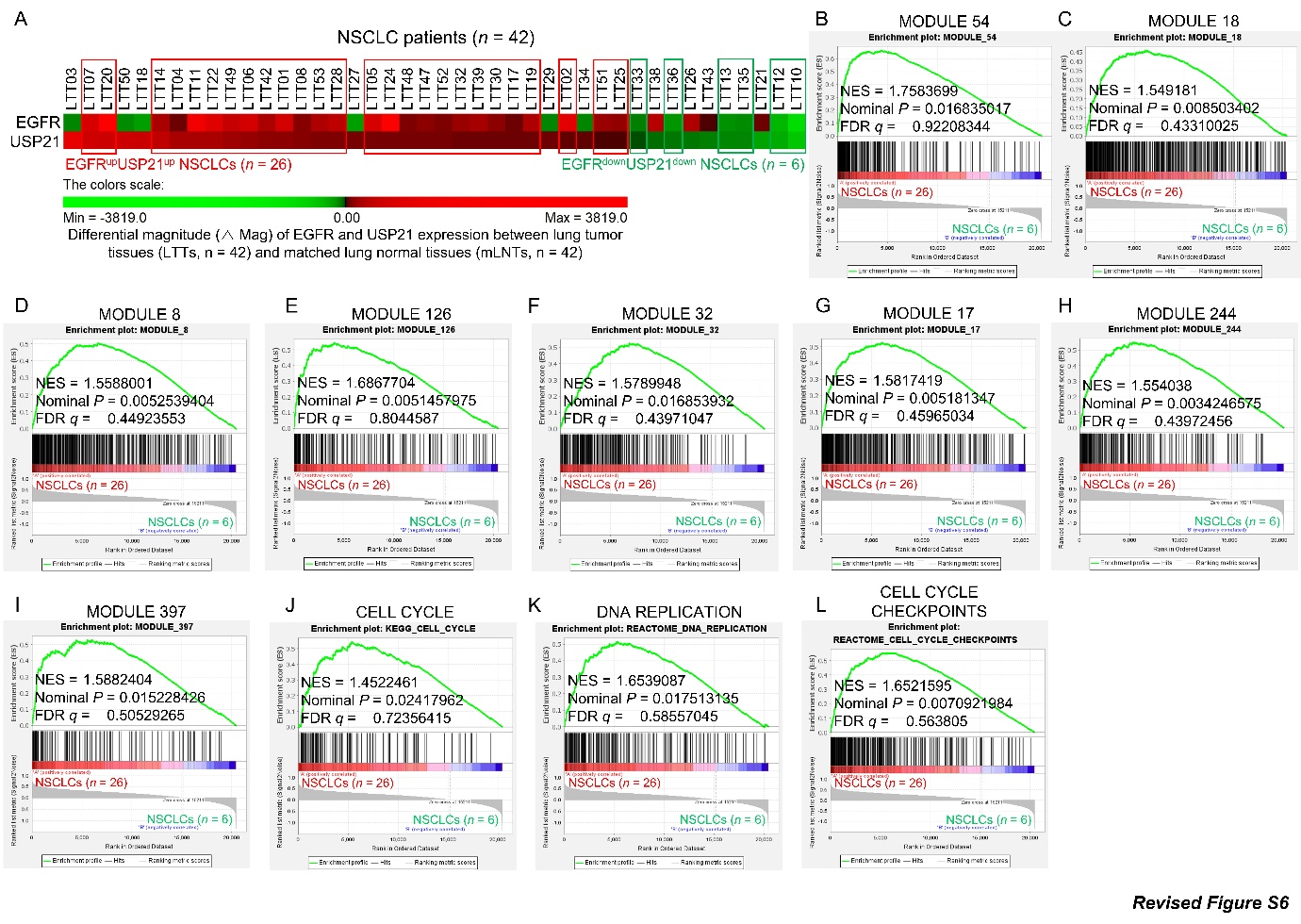
**

**Supplementary Figure S6.** Gene enrichment analysis in NSCLC patients with differential EGFR and USP21 expression. **A** Using microarray data from lung tumor tissues (LTTs, *n* = 42) and matched lung normal tissues (mLNTs, *n* = 42), Δ Mag values for EGFR and USP21 expression (AVG signal) were calculated and ranked. Patients were categorized based on EGFR and USP21 expression levels; EGFR- and USP21-upregulated (EGFR^up^USP21^up^) NSCLC patients (*n* = 26); EGFR- and USP21-downregulated (EGFR^down^USP21^down^) NSCLC patients (*n* = 6) (Table S2). **B-L** GSEA was conducted comparing EGFR^up^USP21^up^ (*n* = 26) and EGFR^down^USP21^down^ (*n* = 6) NSCLC patients. Enriched cancer modules and cell cycle-related genes include: **B**, MODULE 54; **C**, MODULE 18; **D**, MODULE 8; **E**, MODULE 126; **F**, MODULE 32; **G**, MODULE 17; **H**, MODULE 244; **I**, MODULE 397; **J**, CELL CYCLE; **K**, DNA REPLICATION; **L**, CELL CYCLE CHECKPOINTS. Each panel displays the Normalized Enrichment Score (NES), nominal *p*-values, and False Discovery Rate (FDR) q-value.

**
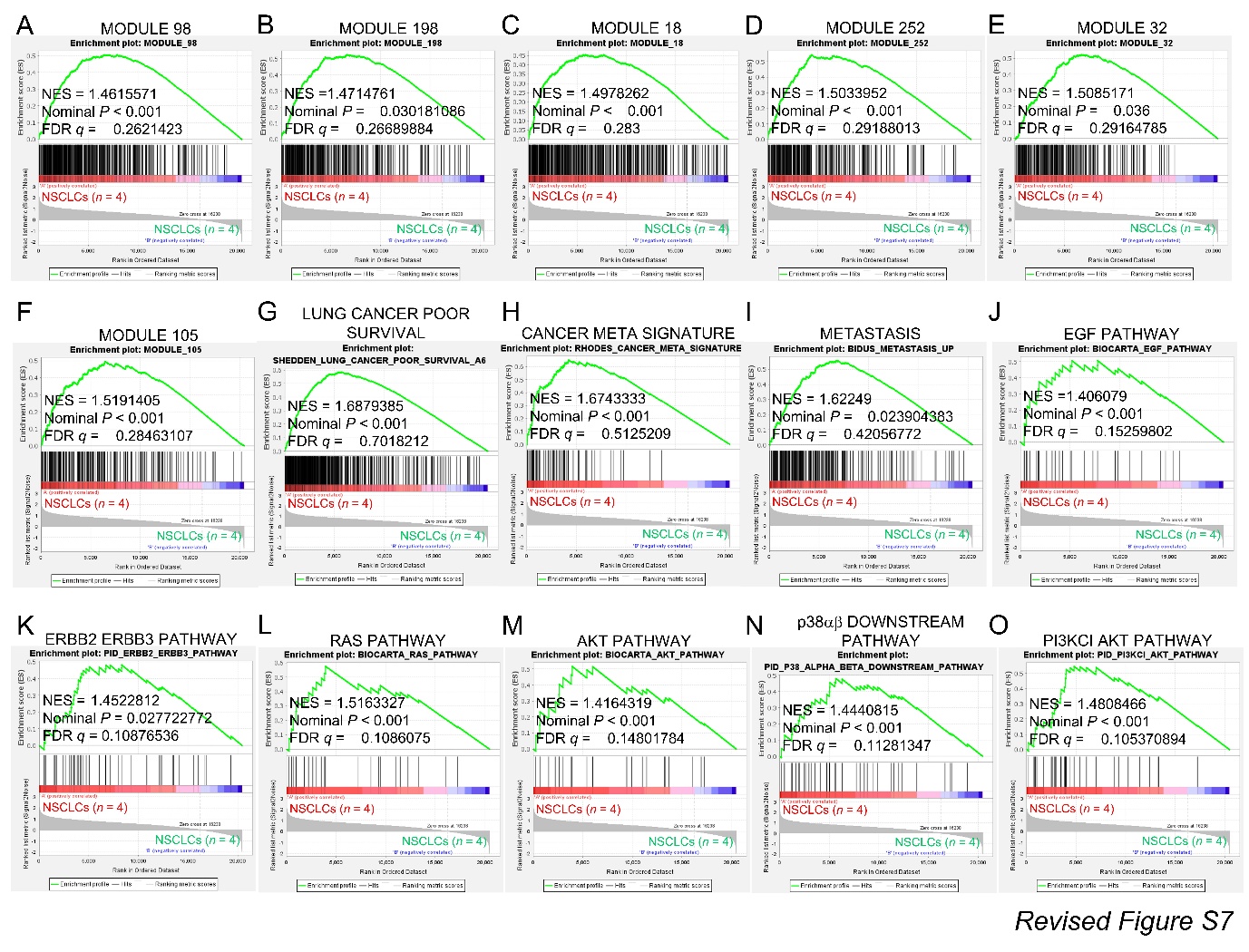
**

**Supplementary Figure S7.** Gene enrichment analysis in NSCLC patients with differential Lyn, EGFR, and USP21 expression. **A-K** Using microarray data from lung tumor tissues (LTTs, *n* = 42) and matched lung normal tissues (mLNTs, *n* = 42), Δ Mag values for Lyn, EGFR, and USP21 expression (AVG signal) were calculated and ranked. Patients were categorized based on Lyn, EGFR, and USP21 expression levels; Lyn-, EGFR-, and USP21-upregulated (Lyn^up^EGFR^up^USP21^up^) NSCLC patients (*n* = 4); Lyn-, EGFR-, and USP21-downregulated (Lyn^down^EGFR^down^USP21^down^) NSCLC patients (*n* = 4) (Table S3). GSEA was conducted comparing Lyn^up^EGFR^up^USP21^up^ (*n* = 4) and Lyn^down^EGFR^down^USP21^down^ (*n* = 4) NSCLC patients. Enriched cancer modules, cell cycle-related, lung cancer and cancer meta signature, and EGFR-related genes include: **A**, MODULE 98; **B**, MODULE 198; **C**, MODULE 18; **D**, MODULE 252; **E**, MODULE 32; **F**, MODULE 105; **G**, LUNG CANCER POOR SURVIVAL; **H**, CANCER META SIGNATURE; **I**, METASTASIS; **J**, EGF PATHWAY; **K**, ERBB2 ERBB3 PATHWAY; **L**, RAS PATHWAY; **M**, AKT PATHWAY; **N**, p38αβ DOWNSTREAM PATHWAY; **O**, PI3KCI AKT PATHWAY. Each panel displays the Normalized Enrichment Score (NES), nominal *p*-value, and False Discovery Rate (FDR) q-value.

**
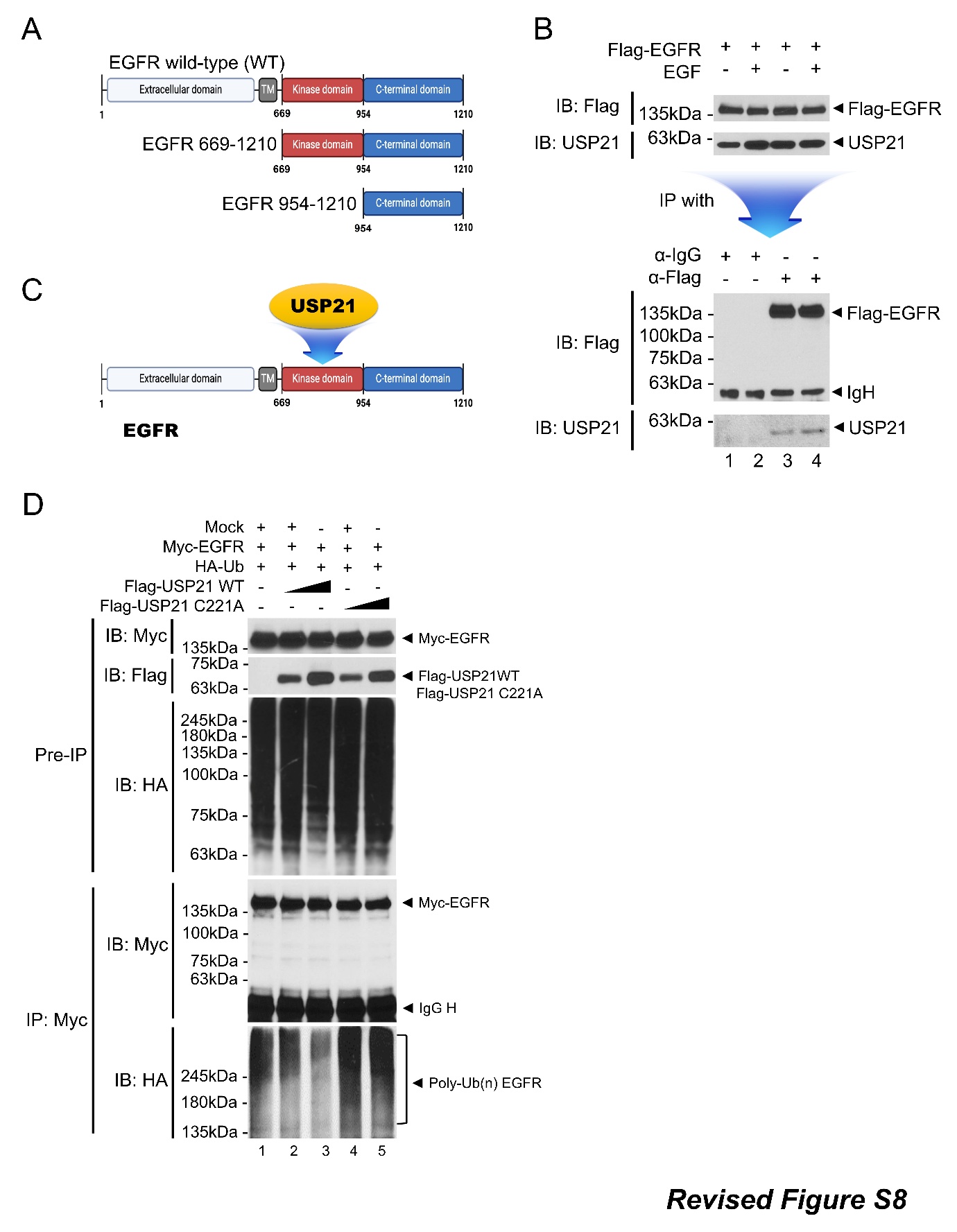
**

**Supplementary Figure S8. A** Truncated mutants of EGFR were generated and represented. **B** H1299 cells were transfected with mock (a control vector) or Flag-EGFR, as indicated. Cells were treated with vehicle (DMSO, 0.1% v/v) or EGF (5 ng/mL) for 30 min. Semi-endogenous immunoprecipitation (IP) assay was performed with anti-IgG or anti-Flag antibody, and immunoblotting (IB) assay was performed with anti-Flag and anti-USP21 antibodies. **C** A model of the interaction between USP21 and EGFR. **D** De-ubiquitination assay with USP21 wild-type (WT) and USP21 C221A mutant vectors. Co-immunoprecipitation (IP) and immunoblotting (IB) analyses in HEK-293T cells transfected with mock vector, Myc-EGFR, HA-Ub, different concentrations of Flag-USP21 wild-type (WT) and Flag-USP21 C221A mutant, as indicated. IP was performed using an anti-Myc antibody, followed by IB with anti-Flag, anti-Myc, or anti-HA antibodies.

**
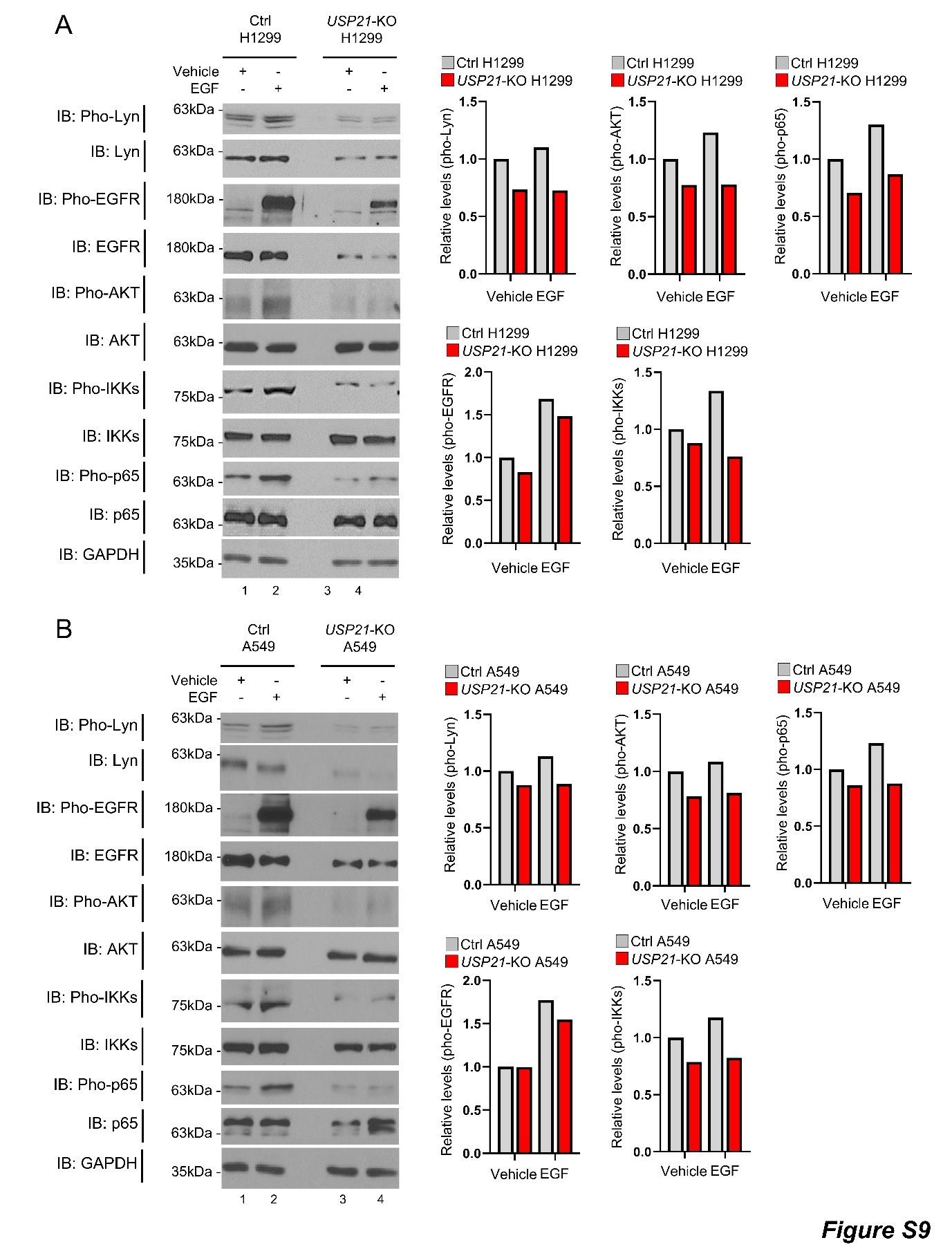
**

**Supplementary Figure S9.** Western blotting analysis in control (Ctrl) and *USP21*-KO lung cancer cells in response to EGF**. A and B** Control (Ctrl) H1299 and *USP21*-KO H1299 cells (**A**) or Ctrl A549 and *USP21*-KO A549 cells (**B**) were seeded into 6-well plates and cultured. Cells were stimulated with vehicle (DMSO, 0.1% v/v) or EGF (5 ng/mL) for 30 min. After collecting cells, cell lysates were separated by sodium dodecyl sulfate-polyacrylamide gel electrophoresis (SDS-PAGE, 8–12 %) and immune-probed with anti-phospho-Lyn, anti-Lyn, anti-phospho-EGFR, anti-EGFR, anti-phospho-AKT1, anti-AKT1, anti-phospho-IKKs, anti-IKKs, anti-phospho-p65, anti-p65, or anti-GAPDH (as loading control) antibodies. Relative levels of phosphorylated proteins, pho-Lyn, pho-EGFR, pho-AKT, pho-IKKs, and pho-p65, were evaluated by using ImageJ software.

**
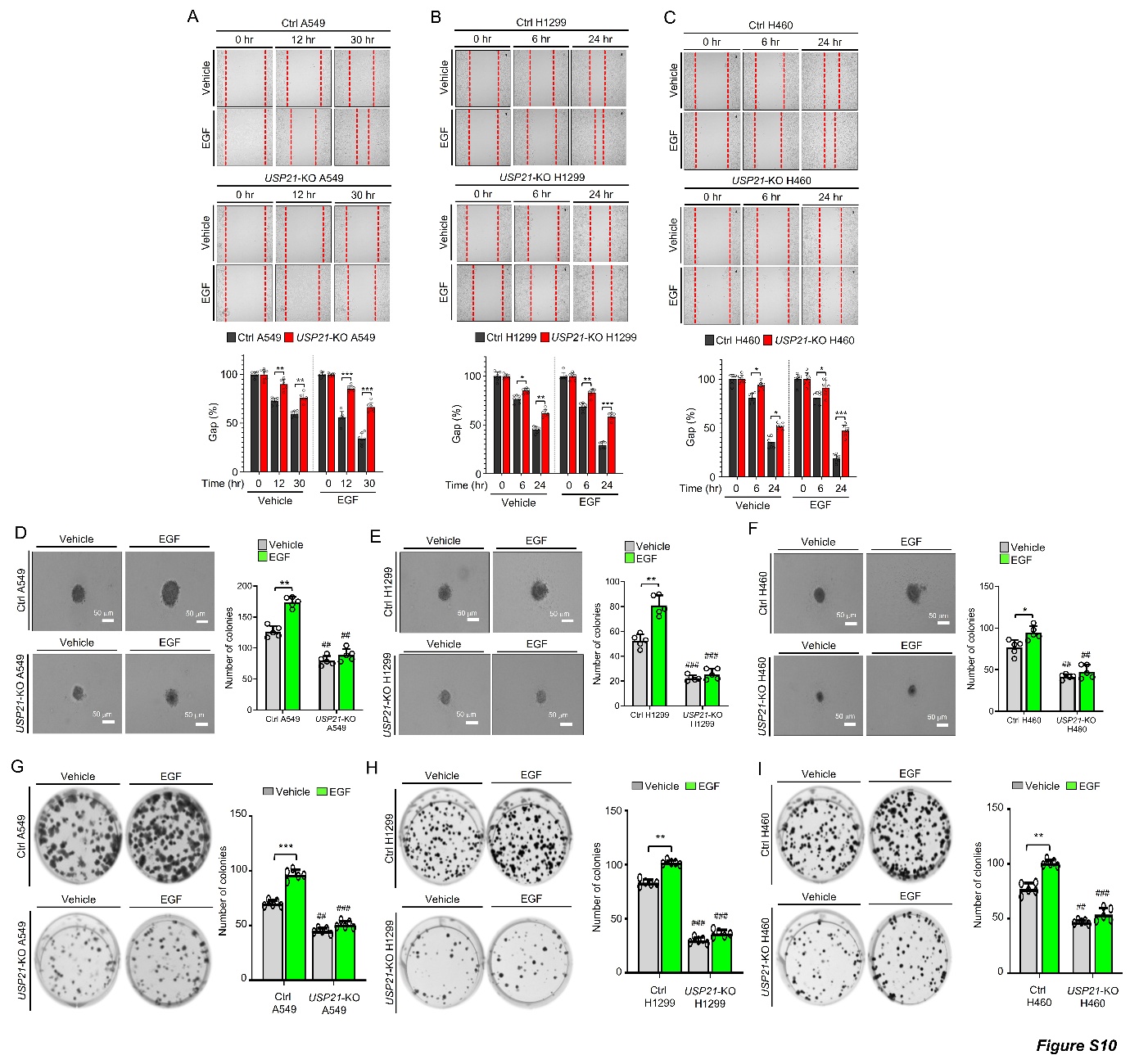
**

**Supplementary Figure S10.** *In vitro* cancer progression assay with *USP21*-knockout (*USP21*-KO) lung cancer cells and control (Ctrl) cells in response to EGF. **A*-*C** Wound healing assays were conducted on Ctrl A549 and *USP21*-KO A549 (**A**), Ctrl H1299 and *USP21*-KO H1299 (**B**), or Ctrl H460 and *USP21*-KO H460 cells (**C**). Cells were treated with vehicle (DMSO, 0.1% v/v) or EGF (15–20 ng/mL) for various time periods. Images of cell migration were captured at indicated time points. Results are presented as mean ± SD (*n* = 5). Statistical significance (Student’s t-test): *, *P* < 0.05; **, *P* < 0.01; ***, *P* < 0.001. **D-F** Anchorage-independent colony formation assays were conducted on Ctrl A549 and *USP21*-KO A549 (**D**), Ctrl H1299 and *USP21*-KO H1299 (**E**), or Ctrl H460 and *USP21*-KO H460 cells (**F**). Cells were treated with vehicle (DMSO, 0.1% v/v) or EGF (15–20 ng/mL) for 30 days. Results are presented as mean ± SD (*n* = 5). Statistical significance (Student’s t-test): *, *P* < 0.05; **, *P* < 0.01. ^##^, *P* < 0.01; ^###^, *P* < 0.001—comparisons between *USP21*-KO cells and their respective Ctrl cells. **G-I** Anchorage-dependent colony formation assays were conducted on Ctrl A549 and *USP21*-KO A549 (**G**), Ctrl H1299 and *USP21*-KO H1299 (**H**), or Ctrl H460 and *USP21*-KO H460 cells (**I**). Cells were treated with vehicle (DMSO, 0.1% v/v) or EGF (15–20 ng/mL) for 9 days. Results are presented as mean ± SD (**G and H**, *n* = 7; **I**, *n* = 5). Statistical significance (Student’s t-test): **, *P* < 0.01; ***, *P* < 0.001. ^##^, *P* < 0.01; ^###^, *P* < 0.001—comparisons between *USP21*-KO cells and their respective Ctrl cells.

**
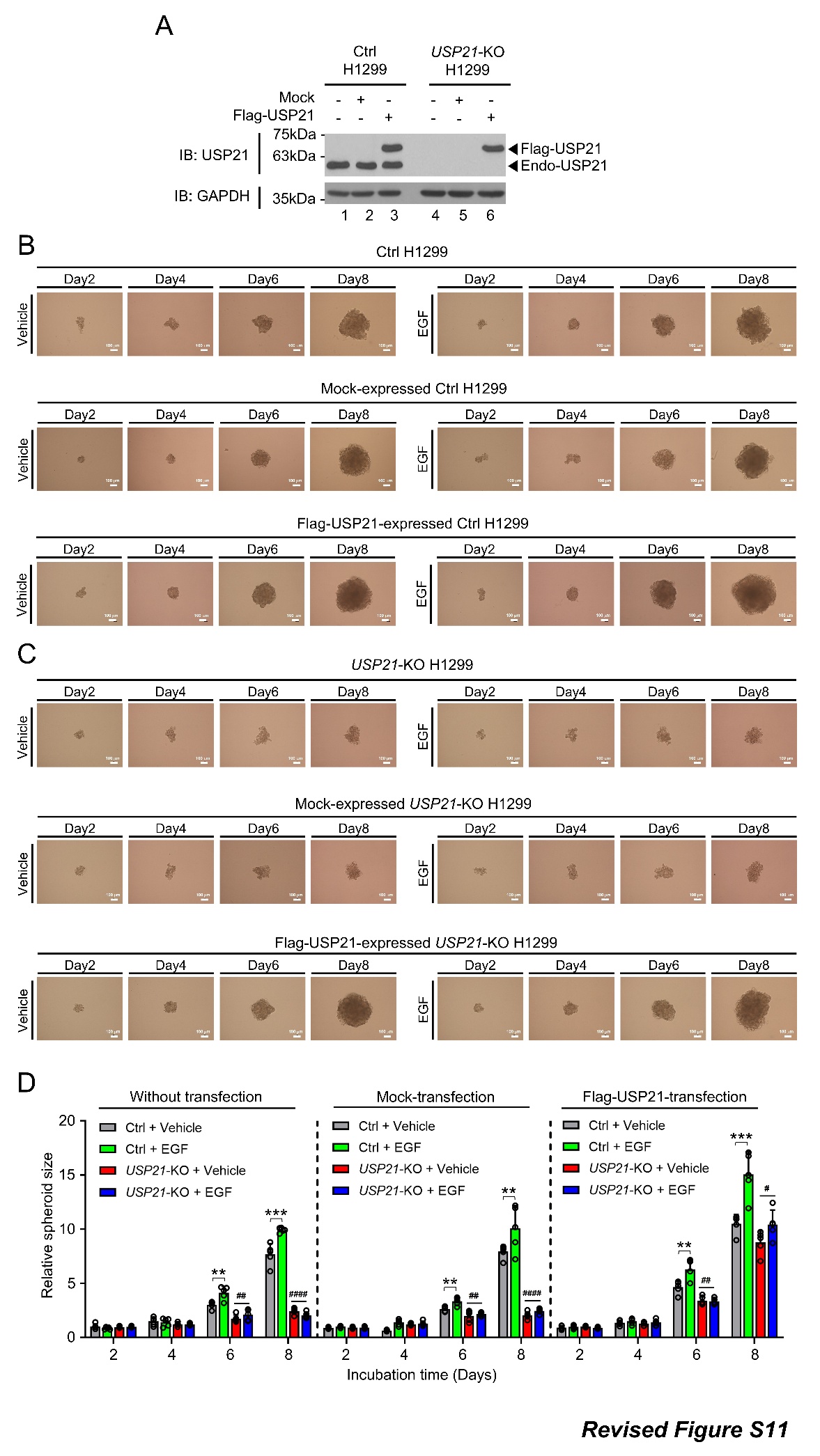
**

**Supplementary Figure S11.** Rescue experiment in *USP21*-KO lung cancer cells transfected with USP21 vector. **A** Ctrl H1299 and *USP21*-KO H1299 cells were transiently transfected with mock (a control vector) or Flag-USP21. Western blotting analysis was performed with anti-USP21 or anti-GAPDH antibody. **B and C** Ctrl H1299 (**B**) and *USP21*-KO H1299 (**C**) cells transfected without, or with mock (a control vector) and Flag-USP21 vector were seeded in 96-well plates and incubated at 37°C for 48 hours to allow spheroid formation. Tumor spheroid formation assay was performed for different times, as indicated. Spheroid size was measured using ImageJ software, and images were captured via phase-contrast microscopy (scale bar = 100 µm). **D** Data are presented as mean ± SD (*n* = 5). Statistical significance (Student’s t-test): **, *P* < 0.01; ***, *P* < 0.001. ^#^, *P* < 0.05; ^#^, *P* < 0.05; ^####^, *P* < 0.0001—comparisons between Ctrl H1299 cells and *USP21*-KO H1299 cells.

**
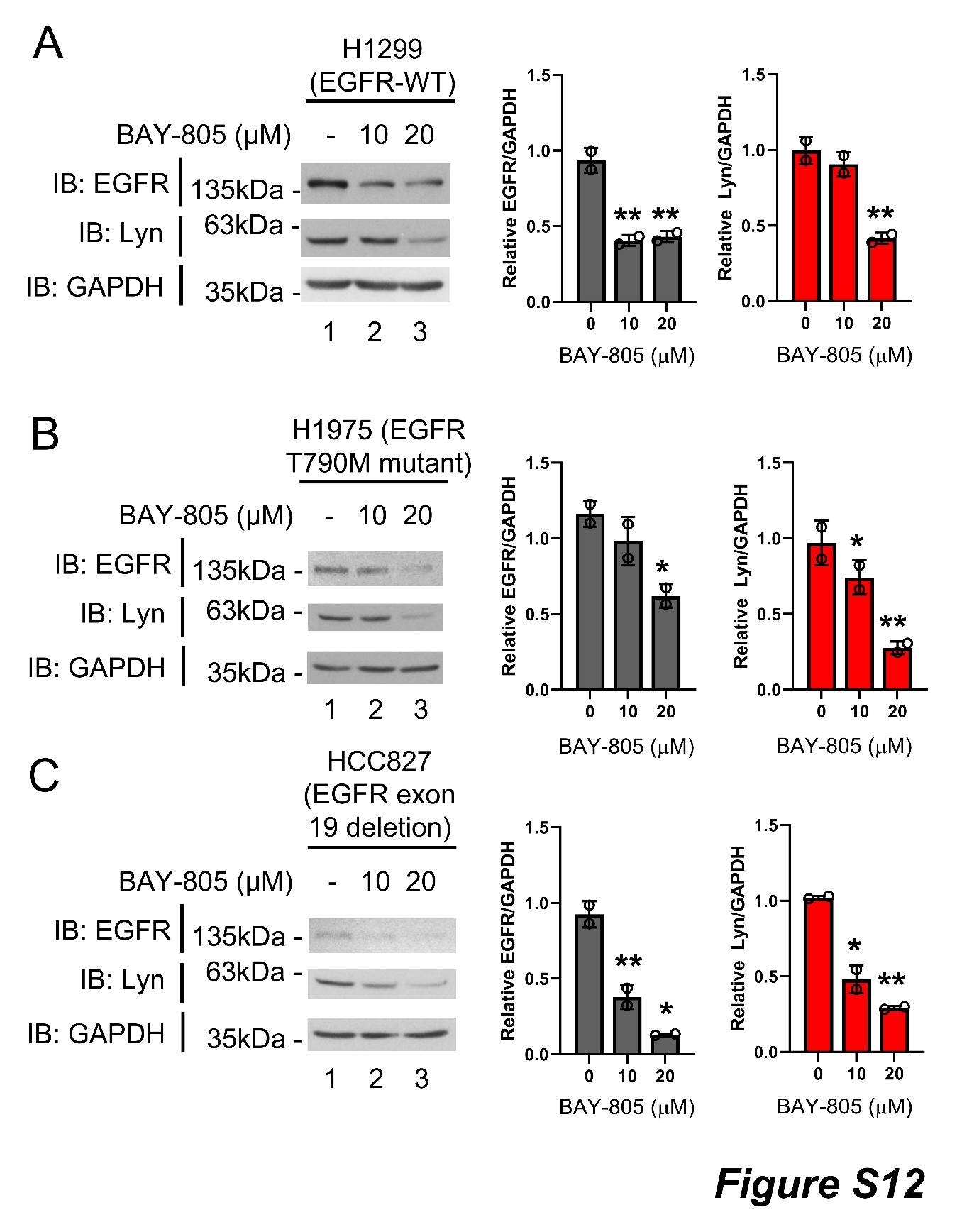
**

**Supplementary Figure S12.** Western blot analysis of EGFR and Lyn expression in EGFR wild-type (WT) and EGFR mutant lung cancer cells treated with BAY-805. **A-C** Western blot analysis of EGFR and Lyn expression in H1299 (EGFR-WT, **A**), H1975 (EGFR T790M mutant, **B**), and HCC827 (EGFR exon 19 deletion, **C**) cells treated with vehicle (DMSO, 0.1% v/v) or different concentrations of BAY-805, as indicated. The levels of EGFR and Lyn expression were quantified relative to GAPDH using ImageJ. Data are presented as mean ± SD (*n* = 3). Statistical significance (Student’s t-test); *, *P* < 0.05; **, *P* < 0.01.

*
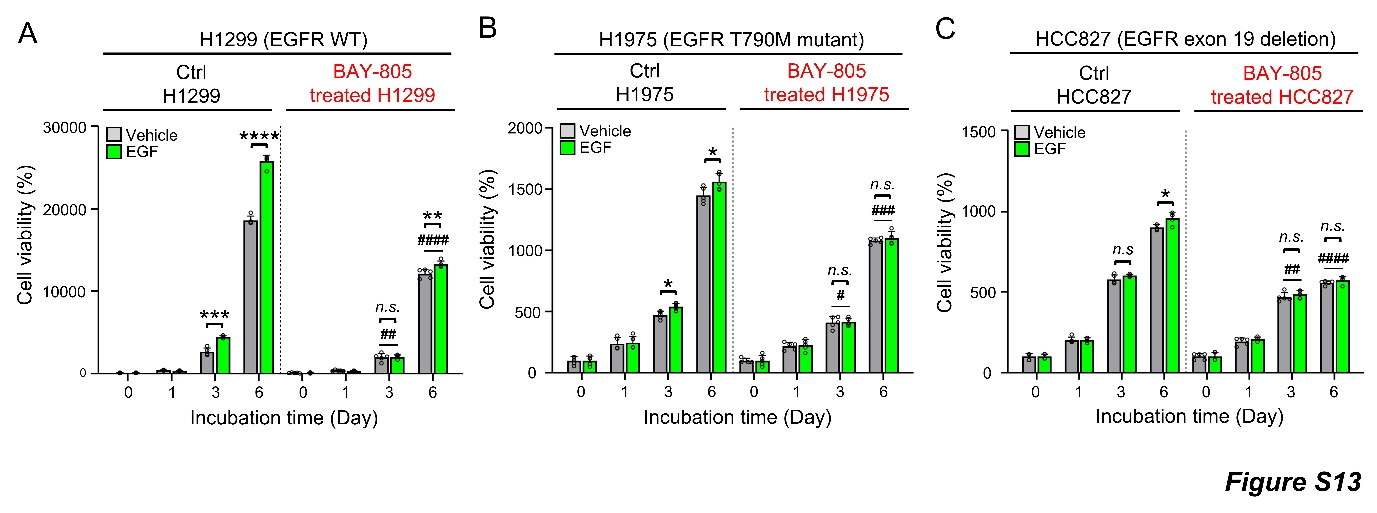
*

**Supplementary Figure S13.** Cell proliferation assay in EGFR wild-type (WT) and EGFR mutant lung cancer cells treated with BAY-805. **A-C** H1299 (EGFR wild-type, **A**), H1975 (EGFR T790M mutant, **B**), and HCC827 (EGFR exon 19 deletion, **C**) lung cancer cell lines were seeded into 96-well plates at a density of 300 cells per well. Cells were treated with either vehicle (0.1% DMSO) or BAY-805 (17 μM) for 24 hours, followed by treatment with or without EGF (10 ng/mL). Cell viability was measured at the indicated time points using the MTT assay. Data are presented as mean ± SD (n = 5). Statistical significance was determined using Student’s t-test: *, *P* < 0.05; **, *P* < 0.01; ***, *P* < 0.001; ****, *P* < 0.0001. ^#^: *P*-values compare vehicle-treated vs. BAY-805-treated cells: ^#^, *P* < 0.05; ^##^, *P* < 0.01; ^###^, *P* < 0.001; ^####^, *P* < 0.0001. *n.s*.: not significant.


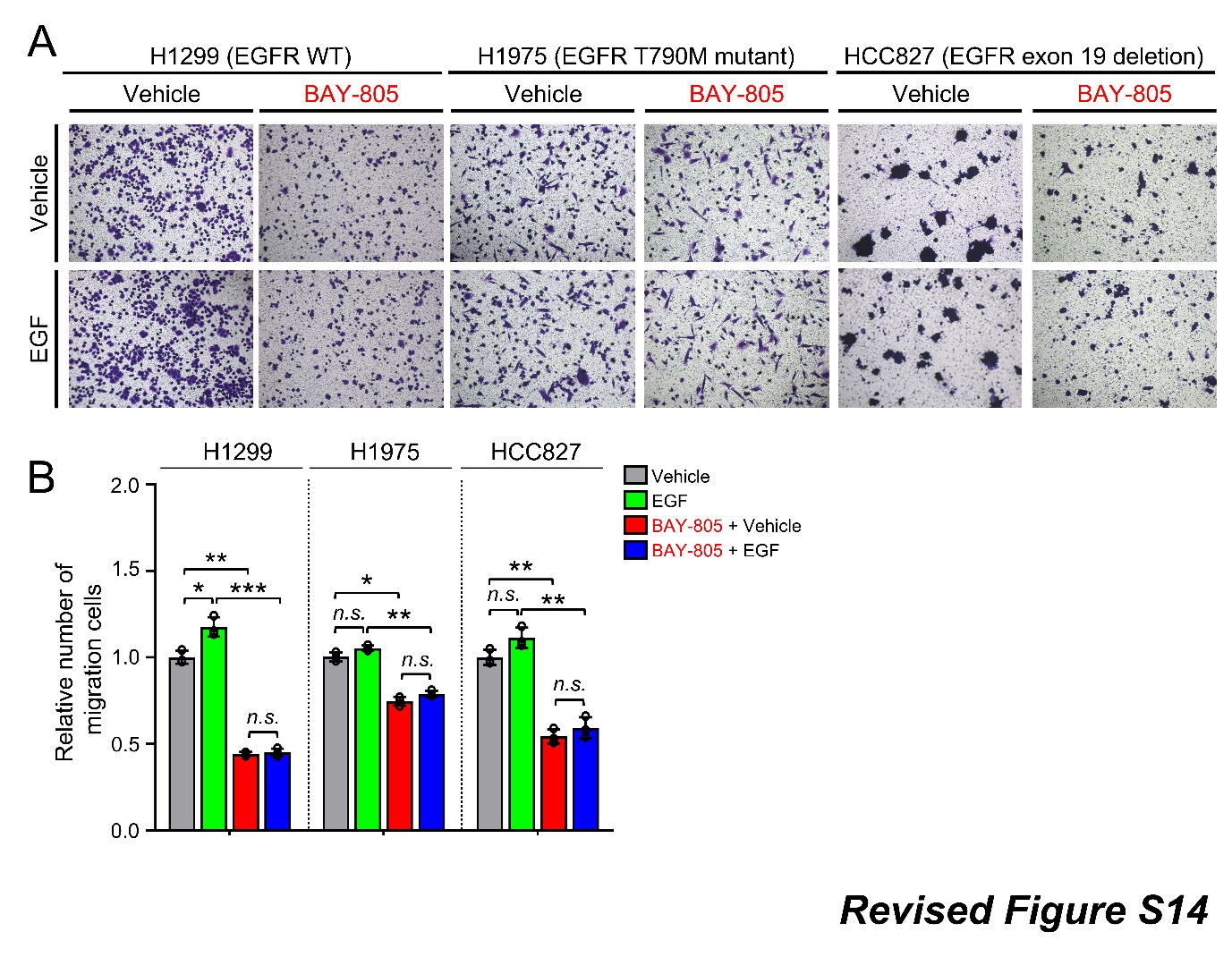


**Supplementary Figure S14.** Transwell migration assay in EGFR wild-type (WT) and EGFR mutant lung cancer cells treated with BAY-805. **A** H1299 (EGFR wild-type), H1975 (EGFR T790M mutant), and HCC827 (EGFR exon 19 deletion) lung cancer cell lines were treated with either vehicle (0.1% DMSO) or BAY-805 (17 μM) for 24 hours. Cells were suspended in 250 μL of culture medium and added to the upper compartment of a 24-well Transwell® chamber (8 μm pore). The cells were treated with vehicle (DMSO, 0.1% v/v) or EGF (10 ng/mL) and incubated at 37 °C for 24 hr. **B** Data are presented as mean ± SD (n = 3). Statistical significance was determined using Student’s t-test: *, *P* < 0.05; **, *P* < 0.01; ***, *P* < 0.001. *n.s*.: not significant.

**
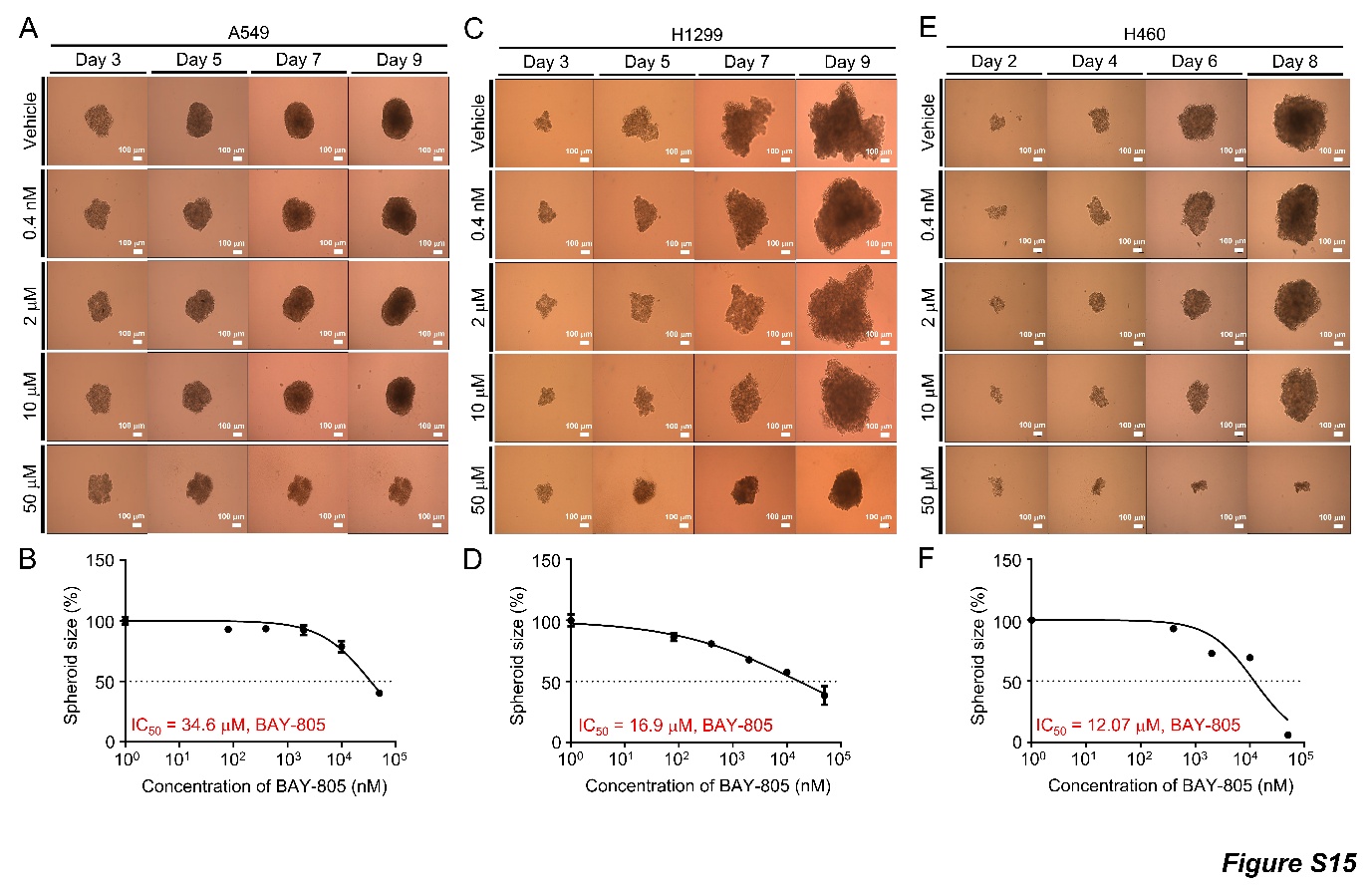
**

**Supplementary Figure S15.** Determination of IC_50_ concentration of BAY-805 on 3D tumor spheroid formation. **A-F** A549 (**A and B**), H1299 (**C and D**), or H460 (**E and F**) lung cancer cells were seeded in 96-well plates at a density of 125 or 500 cells/well and incubated at 37 °C for 48 hours to allow spheroids formation. Spheroids were then treated with vehicle (DMSO, 0.1% v/v) or varying concentrations of BAY-805, ranging from 0.4 nM to 50 μM, and incubated for different time periods. Spheroid size was measured using ImageJ software, and images were captured via phase-contrast microscopy (scale bar = 100 µm). Data are presented as mean ± SD (*n* = 5). The IC_50_ value of BAY-805 was calculated using GraphPad Prism 8.0 software (**B**, A549; **D**, H1299; **F**, H460).

**
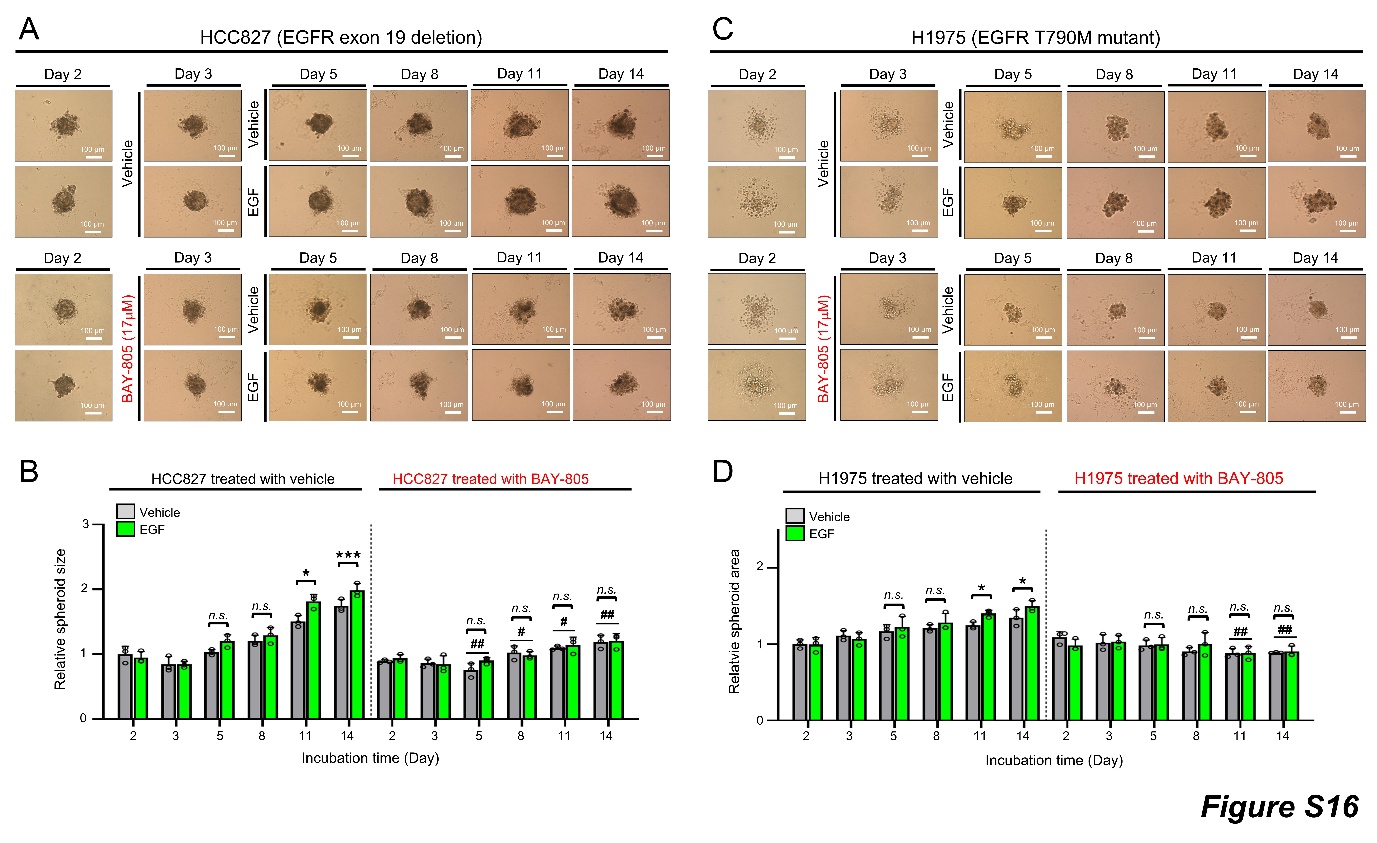
**

**Supplementary Figure S16.** 3D tumor spheroid formation assays were conducted with H1975 (EGFR T790M mutant) and HCC827 (EGFR exon 19 deletion). **A-D** 1.5% agarose hydrogel was added to each well of 96-well culture plates and incubated at room temperature (RT) for 30 min. HCC827 (EGFR exon 19 deletion, **A** and **B**) and H1975 (EGFR T790M mutant, **C** and **D**) cells were seeded into 100 µl growth medium at a density of 250 cells/well. The plates were incubated at 37 °C for 48 hours to allow spheroids formation. Spheroids were pre-treated with either vehicle or 17 µM BAY-805 for 24 hours, followed by treatment with either vehicle or EGF (15 ng/ml). Spheroid size was measured using ImageJ software, and images were captured via phase-contrast microscopy (scale bar = 100 µm). Data are presented as mean ± SD (n = 3). Statistical significance (Student’s t-test): *, P < 0.05; ***, P < 0.001. ^#^, *P* < 0.05; ^##^, *P* < 0.01—comparisons between cells treated with vehicle and with BAY-805. *n.s*.: not significant.

**Supplementary Figure S17.** Raw Western Blot data**.**

**
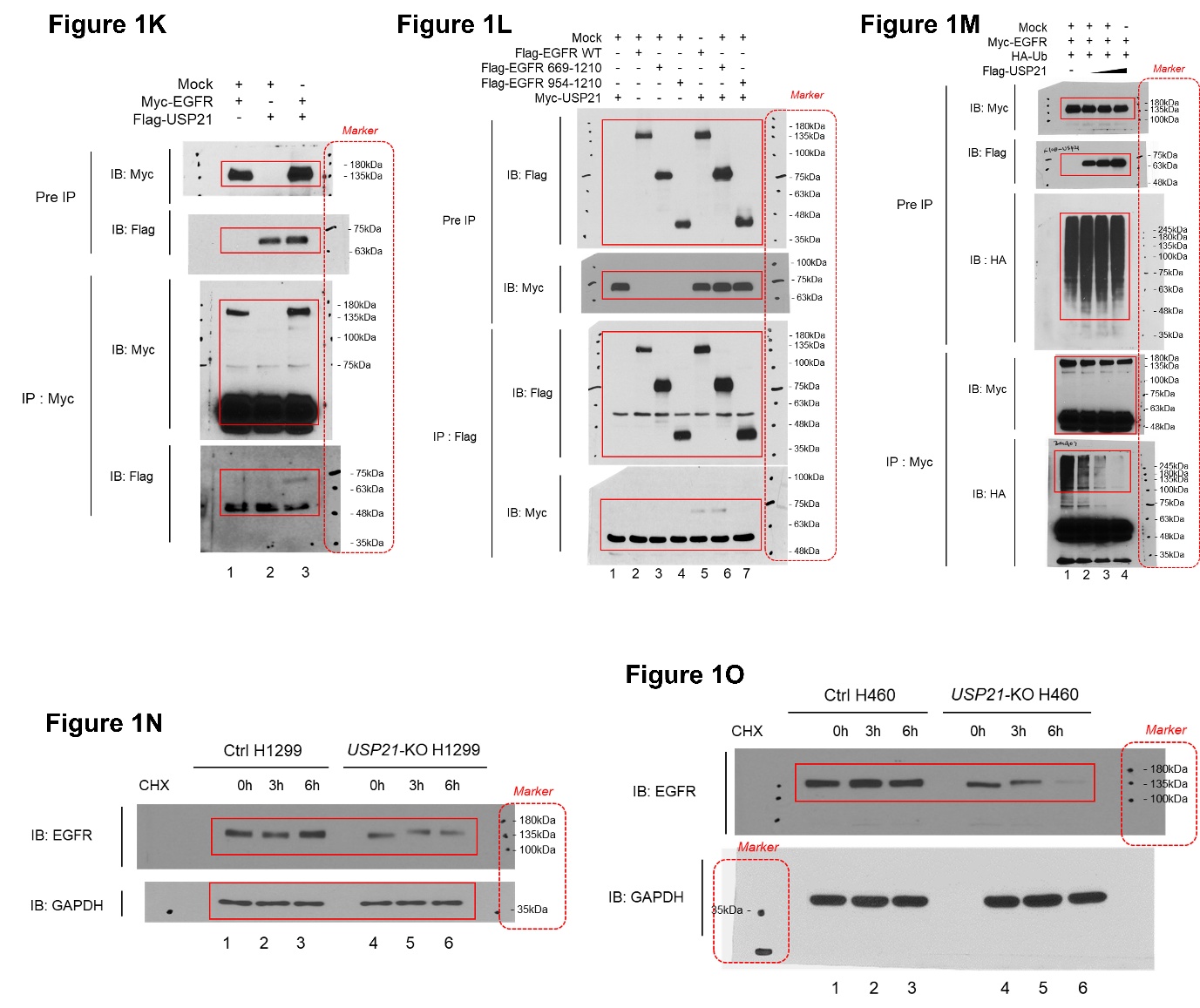
**

**
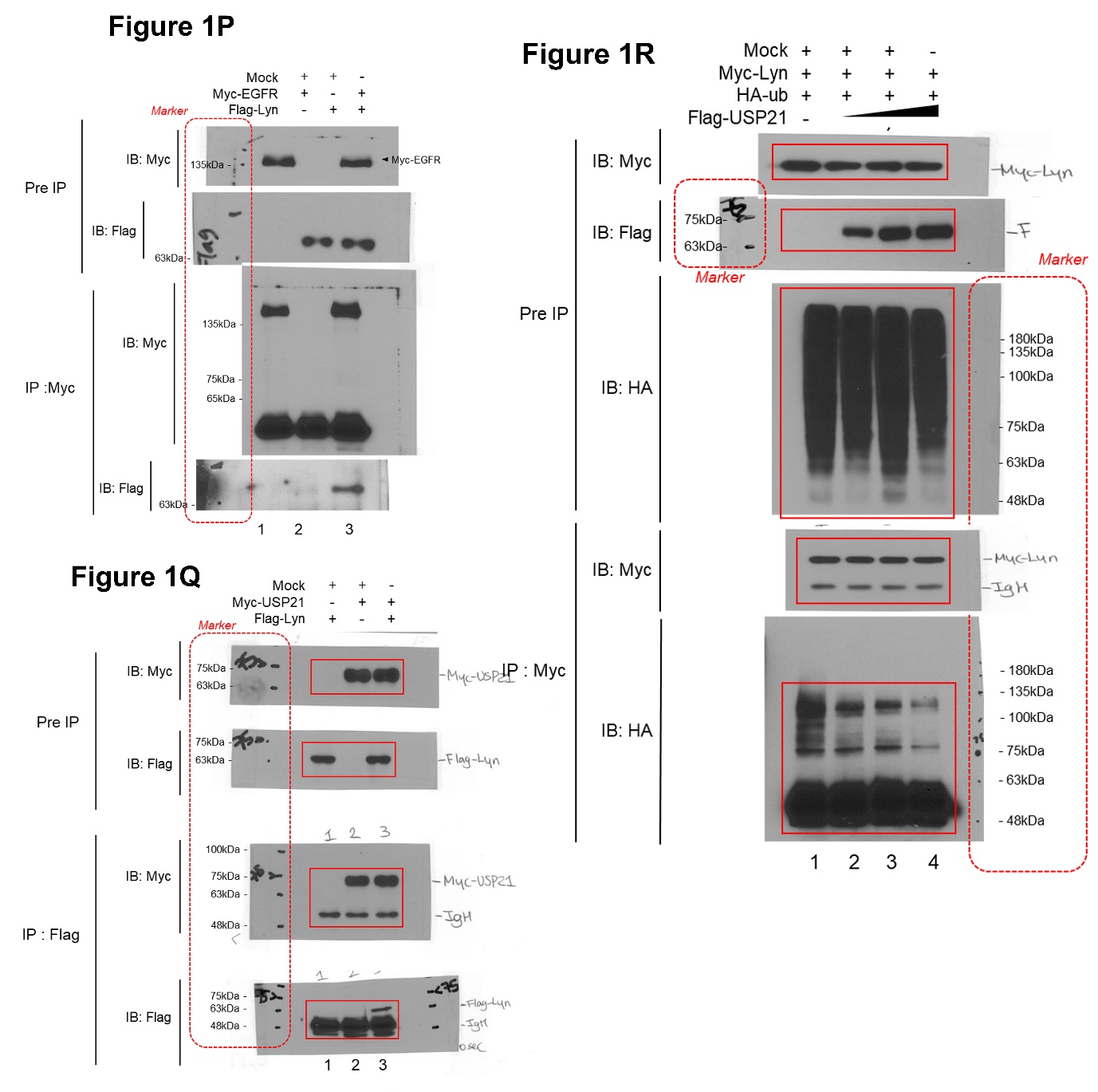
**

**
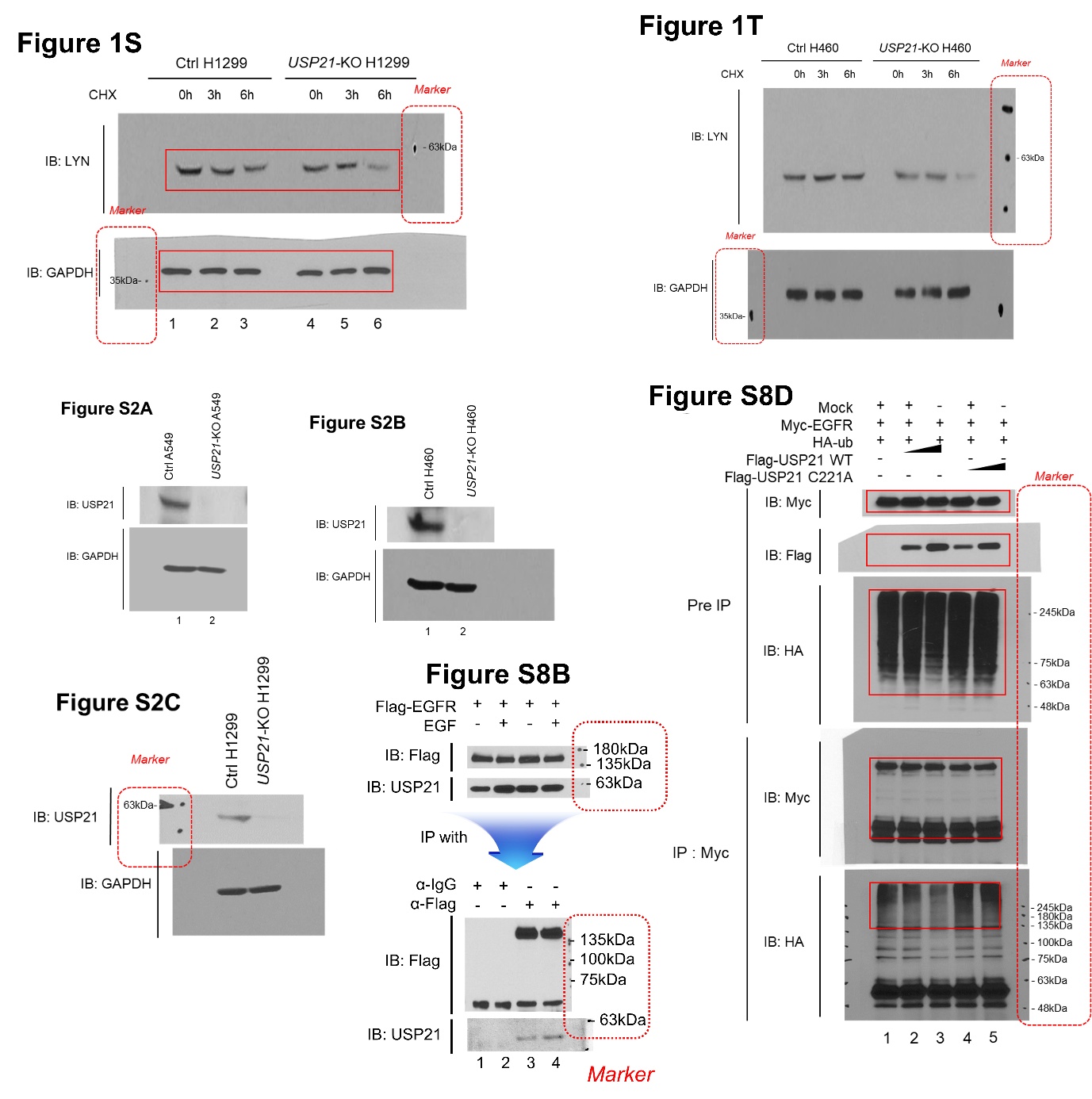
**

**
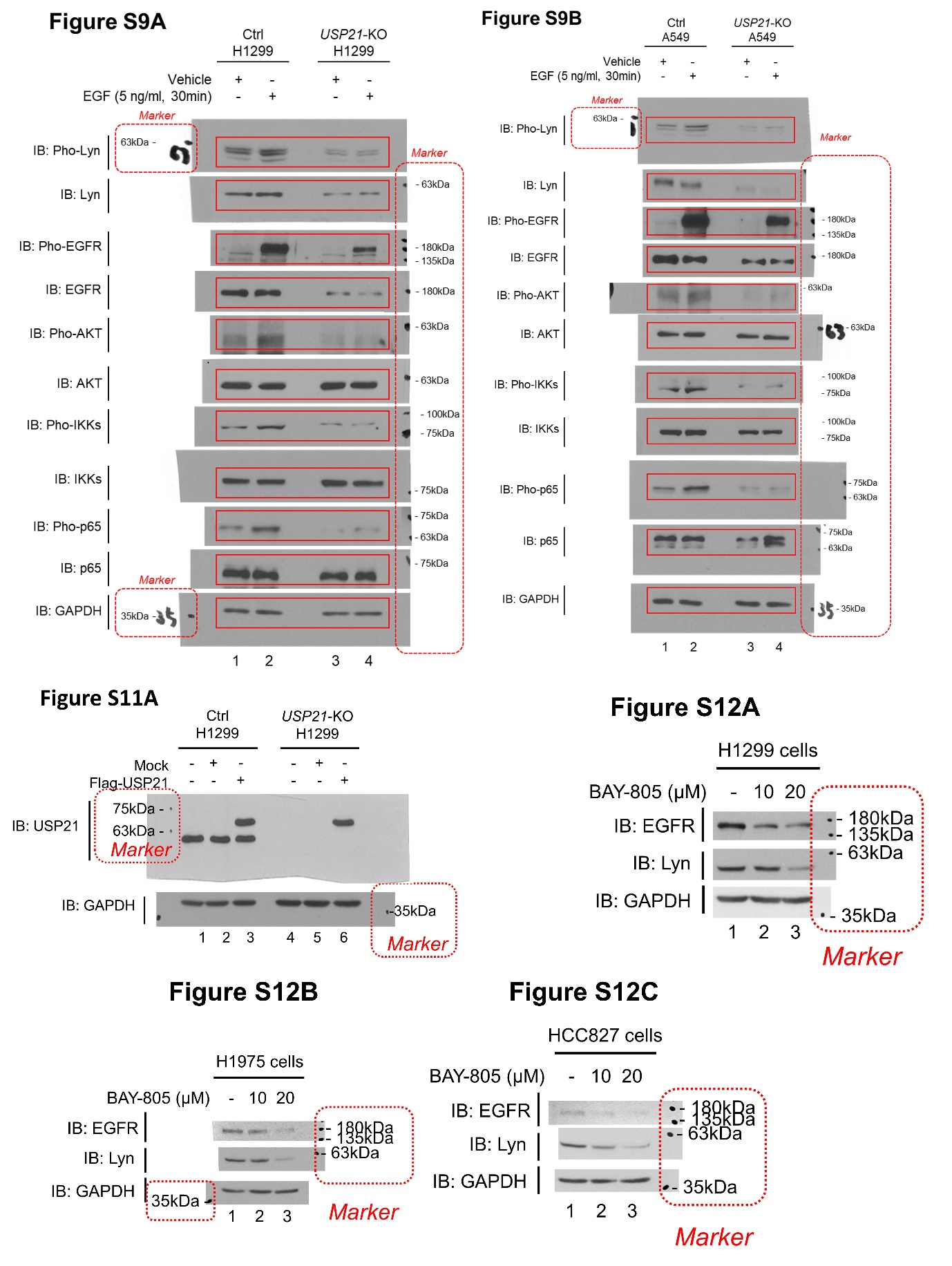
**
